# Switching-On Fluorescence by Copper (II) and Basic Anions: A Case Study with a Pyrene-Functionalized Squaramide †

**DOI:** 10.3390/molecules26051301

**Published:** 2021-02-28

**Authors:** Giacomo Picci, Jessica Milia, Maria Carla Aragoni, Massimiliano Arca, Simon J. Coles, Alessandra Garau, Vito Lippolis, Riccardo Montis, James B. Orton, Claudia Caltagirone

**Affiliations:** 1 Dipartimento di Scienze Chimiche e Geologiche, Università degli Studi di Cagliari, S.S. 554 bivio per Sestu, 09042 Monserrato, Cagliari, Italy; jmilia460@gmail.com (J.M.); aragoni@unica.it (M.C.A.); marca@unica.it (M.A.); agarau@unica.it (A.G.); lippolis@unica.it (V.L.); 2 National Crystallography Service, Department of Chemistry, University of Southampton, Highfield, Southampton SO17 1BJ, UK; S.J.Coles@soton.ac.uk (S.J.C.); J.B.Orton@soton.ac.uk (J.B.O.); 3 Department of Chemical Engineering and Analytical Science, The University of Manchester, Oxford Road, Manchester M13 9PL, UK; riccardo.montis@manchester.ac.uk

**Keywords:** anion recognition, DFT, metal sensing, squaramides, supramolecular chemistry

## Abstract

The new symmetric acyclic *N,N’*-bis(1-pyrenyl) squaramide (**H_2_L**) functionalized with the pyrene moiety as a fluorogenic fragment has been designed and its ability to selectively detect specific anions and metals investigated. **H_2_L** selectively binds Cl^−^ both in solution (DMSO 0.5% H_2_O and MeCN) and in the solid state, and allows to selectively detect Cu^2+^ in MeCN with the formation of a 2:1 metal-receptor complex, with a green intense emission appreciable by naked eye under the UV lamp. The **H_2_L** copper complex preserves its emission properties in the presence of Cl^−^. The addition of basic anions (OH^−^, CN^−^, and F^−^) up to 10 equivalents caused the deprotonation of the squaramide NHs and a dramatic change of the emission properties of the **H_2_L** copper complex.

## 1. Introduction

Squaramides represent an important class of cyclobutene ring derivatives that have stimulated the interest of the scientific community during the last decades, especially in the supramolecular chemistry area. The interest toward this class of compounds arises from their intrinsic features, such as the possibility to act as both H-bond donor and acceptor, the structural rigidity, aromaticity, as well as the ability to form strong bi-directional hydrogen bonds making them ideal candidates for the development of catalysts, self-complementary molecular recognition motifs and self-assembled materials. In particular, these unique properties have also been exploited in the design of squaramide-based systems for anion binding and transport [1,2,3,4] and, recently, as potential low molecular weight gelators and imaging agents [5,6,7].

Notwithstanding their wide application in the field of anion recognition [8,9], to date, only few examples of squaramide based ion pair receptors have been reported [10,11]. Simultaneous complexation of both a cation and an anion by ditopic receptors could provide an enhancement in the binding affinity of the system with respect to simple cation or anion receptors [12,13]. This cooperative binding effect has also been recently confirmed by Romansky and co-workers who developed a family of squaramides functionalized with a crown ether able to bind alkali metal ions and their sulphate counter ion [14].

The use of squaramide-based scaffolds as molecular sensors has also been explored. Several examples of systems enabling detection of the binding event by a colorimetric or fluorometric output has been reported. Starting with the dye-displacement-based sensors, such as those reported by Costa [15], in recent years, a number of different approaches to the design of molecular sensors have been developed [16,17]. The lack of related literature examples that act as efficient molecular sensors highlights the challenge in their design. To the best of our knowledge, from the limited number reported, very few have demonstrated significant responses for the anion sensing. A more promising example was reported by Elmes et al., who described the switch-off response of an anthracene functionalized squaramide, in which the excimer emission of the receptor was switched-off due to the deprotonation of the squaramide –NHs in the presence of Cl^−^ [18]. 

**Scheme 1:**
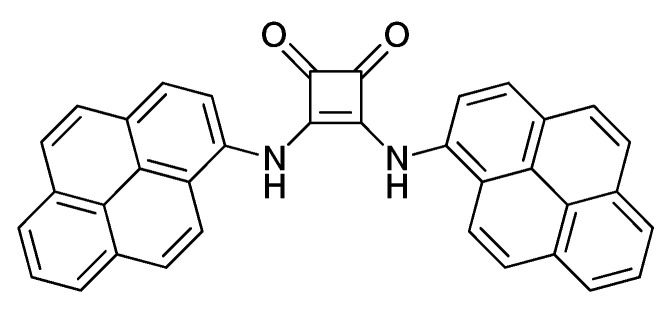
Structure of *N,N’*-bis(1-pyrenyl) squaramide **H_2_L**.

Herein, we report the new symmetric *N,N*’-bis(1-pyrenyl) squaramide (**H_2_L**, Scheme 1) bearing the pyrene moiety as a fluorogenic fragment for anion and metal sensing. The anion binding properties of the receptor towards different anion guests (F^−^, CN^−^, benzoate (BzO^−^), Cl^−^, Br^−^ and I^−^) were studied by means of ^1^H-NMR spectroscopy in DMSO-*d*_6_/0.5% H_2_O and UV-Vis and fluorescence spectroscopies in DMSO/0.5% H_2_O and MeCN, as well as in the solid state. Moreover, in order to assess the ability of **H_2_L** to sense metal ions, we also investigated the metal ion binding properties of **H_2_L** towards different divalent ions (Cd^2+^, Cu^2+^, Hg^2+^, Ni^2+^, Pb^2+^, Zn^2+^) by means of UV-Vis and fluorescence spectroscopies in DMSO/0.5% H_2_O and MeCN. These experimental results were further supported by computational studies.

## 2. Results

### 2.1. Synthesis 

**Scheme 2:**
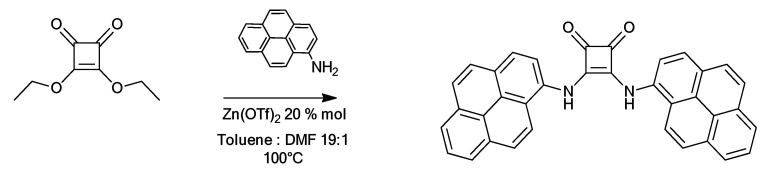
Synthetic pathway for *N,N’*-bis(1-pyrenyl) squaramide **H_2_L**.

**H_2_L** was synthesized using a modified literature procedure [19], by reacting diethyl squarate and 1-amino pyrene (Scheme 2) in a Toluene: DMF: mixture (19:1, *v/v*) in the presence of Zn(OTf)_2_ as a Lewis acid catalyst (see paragraph 3.4 for synthetic details and Figures S1 and S2 for 1H- and 13C-NMR spectra, respectively in the Supplementary Materials (SM)). Single crystals suitable for X-ray diffraction analysis were obtained by slow evaporation of a DMSO solution of the receptor (see Section 2.4).

### 2.2. Anion Binding Studies

Anion-binding studies were conducted by means of ^1^H-NMR titrations using DMSO-*d*_6_/0.5% water as a solvent mixture. Stability constants were determined following the signals of the squaramide NH protons and the signals of the CH protons of the pyrene moiety adjacent to the squaramide NHs upon the addition of increasing amounts of the anions added as tetrabutylammonium (TBA) salts. The obtained ^1^H-NMR titration curves were fitted with a 1:1 binding model using the open source software BindFit [20,21]. The results are summarized in Table 1.

**Figure 1:**
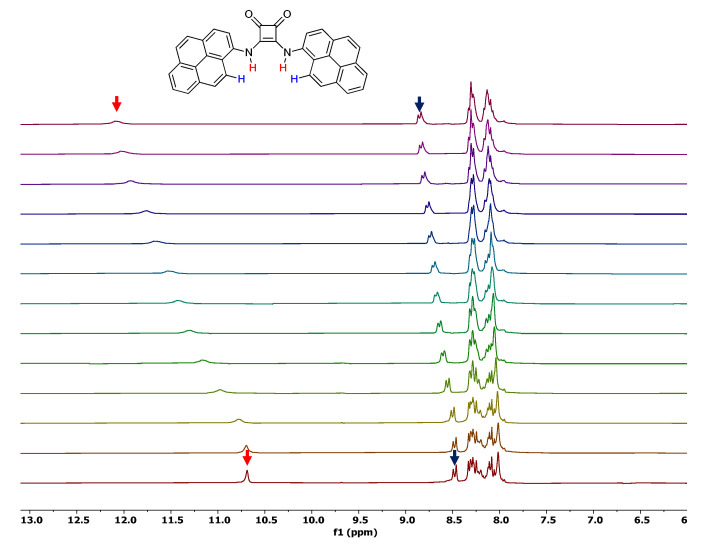
Stack plot of the ^1^H-NMR spectra of **H_2_L** (0.005 M) upon addition of increasing amount of TBACl (0.075 M) in DMSO-*d*_6_/0.5% water.

Under the experimental conditions used, the addition of 0.2 equivalents of F^−^, CN^−^ and BzO^−^ resulted in the disappearance of the ^1^H-NMR signal attributed to the protons of the squaramide NH fragments, accompanied by a dramatic color change of the solution that turned from colorless to yellow (see Figure S3a,b in the SM for the titration with BzO^−^). These observations suggest that **H_2_L** undergoes deprotonation with these anions. On the other hand, **H_2_L** was able to coordinate chloride species both in solution and in the solid state (see Section 2.4). Indeed, upon the addition of increasing amounts of Cl^−^ (as tetrabutylammonium salt) a dramatic downfield shift of the signal attributed to the squaramide NHs (Δδ = 1.4) was observed, as indicated by the red arrow in Figure 1. This was accompanied by a less remarkable downfield shift of the signals attributed to the CHs of the pyrene moiety directly adjacent to the squaramide NHs (Δδ = 0.4) and indicated by the blue arrow in Figure 1. This behavior was attributed to the concomitant formation of a H-bond interaction between the squaramide NHs and the anion guest and to a weaker CH∙∙∙Cl^−^ one that stabilize the formation of the 1:1 anion-receptor adduct. However, the association constant for the formation of the adduct (see Table 1 and Figure S4 in the SM) showed quite low values, indicating that the host-guest molecular recognition process is not very efficient. As suggested by solid-state studies, this might be ascribed to the competition between Cl^−^ and DMSO for the interaction with **H_2_L**. 

The addition of the heavier halides, Br^−^ and I^−^, did not cause any change in the ^1^H-NMR spectrum of the free receptor (Figures S5 and S6 for the 1H-NMR titration of Br− and I−, respectively).

In order to evaluate the anion sensing properties of **H_2_L**, UV-Vis and fluorescence spectroscopies studies were carried out in DMSO/0.5% water and in the less competitive MeCN. We firstly decided to evaluate the effect of the deprotonation on the spectroscopic properties of the receptor upon addition of TBAOH in both media considered. The UV-Vis spectrum of a solution of **H_2_L** in DMSO/0.5% water presents two absorption bands at 280 (ε = 21,500 mol^−1^dm^3^cm^−1^) and 411 nm (ε = 28,200 mol^−1^dm^3^cm^−1^). Upon addition of increasing amounts of TBAOH the disappearance of the band at 411 nm and the formation of two new bands at 348 and 465 nm were observed (see Supplementary Figure S7a in SM). We observed a similar behavior in the presence of CN^−^, and F^−^ (see Supplementary Figure S7b,c, for CN−, and F−, respectively) confirming the results obtained by ^1^H-NMR titrations. Interestingly, the addition of increasing amount of BzO^−^ caused a gradual change of the absorption spectrum. A bathochromic shift of 6 nm of the absorption band at 411 nm and the formation of a new band at 472 nm with the appearance of an isosbestic point at 430 nm (see Figure S7d in the SM) was observed; in the case of the titration with Cl^-^, however, no significant changes in the UV-Vis spectrum of the receptor was observed (see Figure S7e), suggesting a concentration dependence of the host-guest interaction for both anions [22].

**Figure 2:**
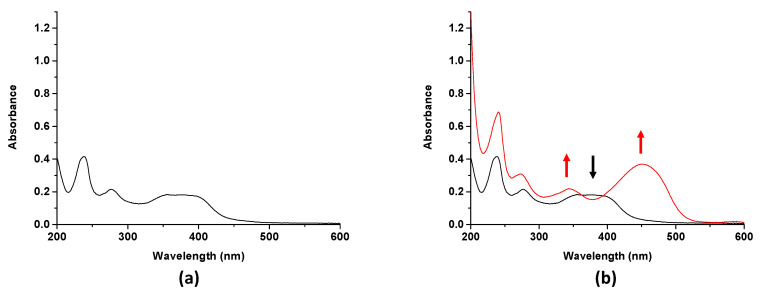
(**a**) Absorption spectra of **H_2_L** (1.0 × 10^–5^ M) in MeCN; (**b**) Absorption spectrum of **H_2_L** as a free receptor (black line) and upon the addition of 8 equivalents of TBAOH (2.5 × 10^−3^ M) (red line).

The UV-Vis absorption spectrum of a solution of **H_2_L** in MeCN solution presents a broad band at 365 nm (ε = 17,800 mol^−1^dm^3^cm^−1^) and other two bands at 276 (ε = 20,700 mol^−1^dm^3^cm^−1^) and 238 nm (ε = 41,400 mol^−1^dm^3^cm^−1^), respectively (Figure 2a). 

As shown in Figure 2b and Figure S8a, upon the addition of increasing amounts of TBAOH we observed the disappearance of the band centered at 365 nm, with the formation of two new absorption bands centered at 343 nm and 452 nm accompanied by a visible color change of the solution from colorless to yellow. We observed the same behavior in the presence of CN^−^ and F^−^ (see Figure S8b,c in the SM for CN^−^ and F^−^, respectively). The results obtained with BzO^−^ (see Figure S8d) closely resembles those previously described, confirming the formation of an adduct with this anion at the concentration used for UV-Vis measurements. The changes in the UV-Vis spectrum of **H_2_L** upon addition of Cl^−^ were similar to those observed in the titration with BzO^−^ (compare Figure S8d,e in SM). As expected, in MeCN, a less competitive solvent with respect to DMSO/0.5% H_2_O, the formation of the **H_2_L**-Cl^−^ adduct could be detected. 

With these results in mind, we decided to perform fluorescence titrations in MeCN with the set of anions tested (See Figure S9). **H_2_L** shows a low intensity emission band centered at 425 nm (Φ = 0.015) when excited at 365 nm (corresponding to the maximum absorption band of the receptor in this medium) ascribable to the pyrene monomer emission. Under the experimental conditions used, upon the addition of increasing amount of OH^−^, no significant changes in the emission pattern of **H_2_L** were observed (see Figure S9a), suggesting that the emission properties of the receptor were not affected by its deprotonation. Curiously, upon the addition of F^−^ and CN^−^ a particular behavior was observed (see Figure S9b,c for CN^−^ and F^−^, respectively). Indeed, upon the addition of less than one equivalent of the guest, we observed the formation of a new emission band centered at 485 nm. This band immediately disappeared with the addition of one equivalent of the two anions in solution, suggesting that a strong interaction with the anions occurs before the deprotonation. No significant changes were observed in the presence of BzO^−^ (see Figure S9d), while the addition of increasing amount of Cl^-^ caused the shift of the emission band of **H_2_L**, from 425 to 461 nm (see Figure S9e). 

The results discussed above suggest that the scarce response of this receptor as fluorescent molecular sensor for anions may well result from the significant steric hindrance present in this molecule. The simultaneous presence of both N-H and carbonyl groups on the squaramide core, causes a decrease in the free rotation around the C-N bond [23], along with the significant steric hindrance due to the presence of the pyrene moieties as a substituent. Indeed, we have previously observed that the introduction of flexible spacers between the fluorophore and the squaramide core enhances the fluorescence response of the system [4].

### 2.3. Metal Ion Sensing by **H_2_L**: Spectrophotometric Measurements

**Figure 3:**
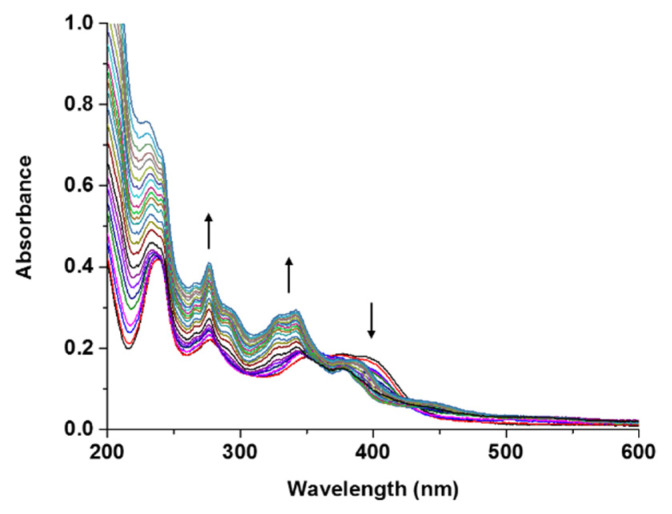
Absorption changes of **H_2_L** (1.0 × 10^−5^ M) spectra upon the addition of increasing amount of Cu(ClO_4_)_2_ hydrate (2.5 × 10^−3^ M) in MeCN.

In order to analyze the ability of **H_2_L** as a fluorescent molecular sensor for metal ions, spectrophotometric and spectrofluorimetric studies of **H_2_L** towards several metal ions (Cd^2+^, Cu^2+^, Hg^2+^, Ni^2+^, Pb^2+^, and Zn^2+^ as nitrate or perchlorate salts) were carried out (see Figure S10). The set of the metal ions was selected considering their biological relevance (Cu^2+^ and Zn^2+^) or their high toxicity (Cd^2+^, Hg^2+^, Ni^2+^, and Pb^2+^). We only observed significant changes in the UV-Vis spectrum of **H_2_L** in MeCN upon the addition of Cu^2+^ ions. Most notably, the band at 365 nm of the free receptor decreased, along with the appearance new bands at 330 nm, 341 nm, and 375 nm (Figure 3). 

**Figure 4:**
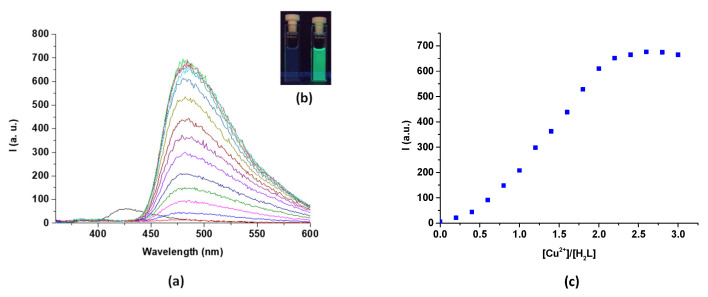
(**a**) fluorescent spectra for the spectrofluorimetric titration in MeCN of **H_2_L** (1.0 × 10^−5^ M) in the presence of increasing amount of Cu(ClO_4_)_2_ hydrate (2.5 × 10^−3^ M) λ_exc_ = 350 nm; (**b**) color change of **H_2_L** upon the addition of 2 equivalents of Cu(ClO_4_)_2_ hydrate; (**c**) fluorescence intensity at 480 nm vs. molar ratio plot for the titration of **H_2_L** with Cu(ClO_4_)_2_ hydrate.

We also observed a selectivity response towards Cu^2+^ by fluorescence. Indeed, as shown in Figure 4a, upon the addition of 0.2 equivalents of Cu^2+^ to a MeCN solution of the receptor the emission band centered at 425 nm switched off with the concomitant formation of a new band centered at 480 nm. The intensity of the new band increased, reaching the maximum upon the addition of about 2.0 equivalents of Cu^2+^ ion, appreciable by naked eye under the UV lamp (Figure 4b) with a green intense emission (Φ = 0.073). The inflection point in the fluorescence intensity/molar ratio plot (Figure 4c) suggested the formation in solution of a 2:1 metal-to-ligand complex. The formation of the 2:1 metal-to-ligand complex was confirmed by fitting the UV-Vis titration data using the open source software BindFit [20,21]. Indeed, a stability constant of Log *K*_1:2_ = 7.2 was calculated (see SI Figure S13).

**Figure 5:**
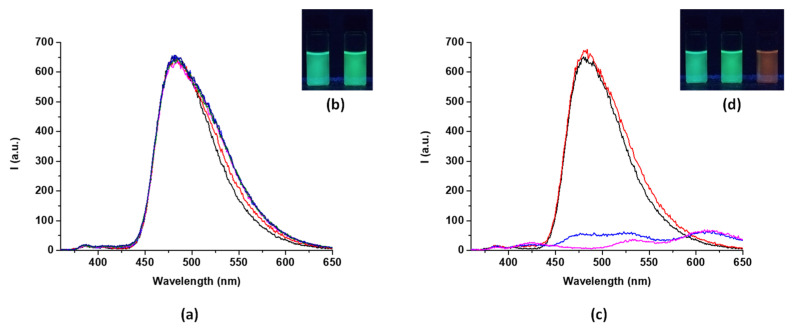
(**a**) spectrofluorimetric changes in MeCN of **H_2_L**-copper complex (2.5 × 10^−5^ M) upon the addition of increasing amount of TBACl (2.5 × 10^−3^ M) up to 10 equivalents, λ_exc_ = 350 nm; (**b**) naked eye emission change of copper complex of **H_2_L** before (left) and after (right) the addition of 10 equivalents of TBACl; (**c**) spectrofluorimetric changes of copper complex of **H_2_L** upon the addition of increasing amount of TBAOH (2.5 × 10^−3^ M) up to 6 equivalents, λ_exc_ = 350 nm; (**d**) naked eye emission change of copper complex of **H_2_L** before (left) and after the addition of 2 equivalents (middle) and 6 equivalents (right) of TBAOH.

Encouraged by these results, we decided to further investigate the fluorescence response of the copper-**H_2_L** complex upon addition of anions. Increasing amounts of OH^−^, CN^−^, F^−^, and Cl^−^ were added to a MeCN solution of the preformed copper(II) complex and the changes in the fluorescence emission recorded. No changes in the emission properties of the complex in the presence of an excess of Cl^−^ (Figure 5a,b) were observed. An interesting behavior was observed in the presence of more basic anions, such as OH^−^, CN^−^, and F^−^. Indeed, the addition of two equivalents of these anions did not affect the emission of the copper complex. In this context, the presence of an excess (up to 4 equivalents) caused the quenching of the emission band, along with a weak bathochromic effect (Figure 5c for OH^−^ and Figure S11a,b for CN^−^ and F^−^, respectively). This was also accompanied by a dramatic change of the emission appreciable at naked eye under a UV lamp that turned from green to pale red (Figure 5d). This experimental evidence might be correlated to the deprotonation of the squaramides NHs in the copper complex. In order to confirm that the addition of the basic anions did not cause the decomplexation of the receptor the same experiments have been conducted by means of UV-Vis spectroscopy. As shown in Figure S12, the addition of 2.0 equivalents of the anion species did not cause significant changes in the absorption spectrum of the copper-**H_2_L** complex. Interestingly, further addition of the more basic anions up to 10 equivalents (OH^−^, CN^−^, and F^−^, Figure S12a–c) caused the decrease of the absorption band at around 380 nm with the concomitant formation of a new band centered at around 480 nm similar to that observed for the deprotonation of **H_2_L**. These observations suggest that the variations of the emission properties are probably due to the deprotonation of the squaramide NHs of the complex. Finally, the addition of TBACl did not cause significant changes in the absorption spectrum of the copper-**H_2_L** complex, corroborating the hypothesis of the deprotonation of the complex as the cause of the changes observed in the fluorescence emission. 

### 2.4. Solid State Studies

Solid state studies are consistent with the behavior observed in solution. In particular, crystallizations of **H_2_L** in the presence of various anions, produced single crystals only with an excess of TBACl, confirming the affinity of **H_2_L** toward the Cl^−^ anion. Although we performed various crystallization attempts in the presence of various anions and metals, structures neither of the deprotonated receptor, nor of any metal complex could be identified during this study. Crystallization of **H_2_L** from DMSO produced a solvate form of the receptor **H_2_L**∙ 2DMSO (**A**). Crystallographic parameters and main intermolecular interactions are shown in Tables S1 and S2 (reported in SM) respectively.

**Figure 6:**
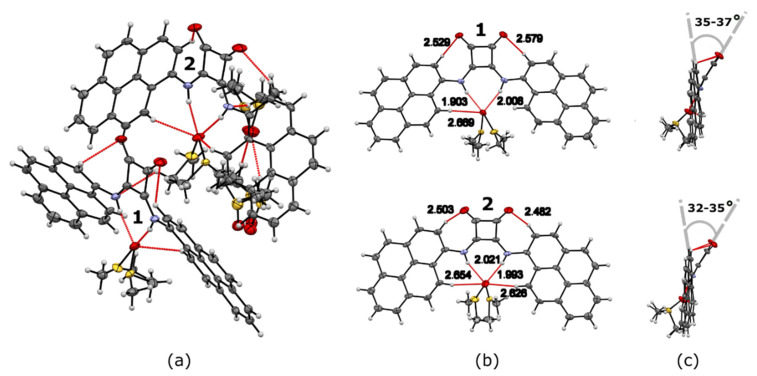
Crystal structure of **H_2_L**∙ 2 DMSO (**A**). (**a**) Asymmetric unit of **A**; (**b**) crystallographically independent receptor units **1** and **2** and main host-guest intermolecular interactions viewed along a direction perpendicular to the plane of pyrene rings; (**c**) symmetrically independent receptor units **1** and **2** viewed along a direction parallel to the plane of pyrene rings. The range of values of interplanar angles between the squaramide cyclobutene plane and the pyrene planes is also showed. Thermal ellipsoids drawn at the 50% probability level.

Yellow lath-shaped single crystals of **H_2_L** ∙ 2DMSO (**A**) were obtained by slow evaporation from a solution of the pure receptor in DMSO, and analyzed by single-crystal X-ray diffraction. The structure of **A** crystallized into the monoclinic crystal system (space group *P2_1_/n*) with two symmetrically independent **H_2_L** molecules and four disordered solvent molecules (Z’ = 2) in the asymmetric unit (Figure 6a). Each receptor unit adopts a non-planar conformation, with angles between the plane of the cyclobutene spacer and the plane of the pyrene pendant arms in the range of 32–37° (see Figure 6c). The structure shows a set of weak intramolecular C-H∙∙∙O hydrogen bonds (H∙∙∙O distances are in the range 2.48–2.58 Å), involving the squaramide C=O groups and the pyrene CHs adjacent to the squaramide NH (see Figure 6b). In each symmetrically independent receptor molecule, the pseudo-cavity is occupied by one solvent molecule, disordered over two positions and interacting via set of N-H∙∙∙O hydrogen bonds (H∙∙∙O distances are 2.01(4)Å and 1.90(3) Å for molecule 1 and 2.02(4) Å and 1.99(4) Å for molecule 2).

**Figure 7:**
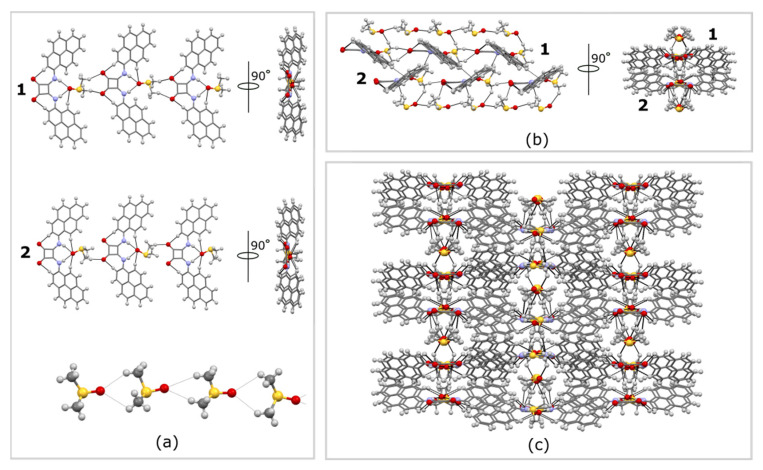
Crystal structure of **A**. (**a**) 1-D chains of receptors 1, 2 and DMSO molecules, viewed down two perpendicular directions; (**b**) Inter-connections of different 1-D chains along the (10$\bar{1}$) directions viewed down two perpendicular directions; (**c**) crystal packing of **A** viewed down the (101) direction. Symmetrically independent receptor units are indicated as **1** and **2**, disorder has been removed for clarity.

The remaining two independent solvent molecules interact to each other via sets of C-H∙∙∙O contacts (H∙∙∙O distances are in the range 2.47–2.65 Å) involving the methyl groups and the S=O acceptors, to form infinite chains that develop along the (101) direction of the unit cell (Figure 7a). 1-D solvent chains are a common feature, and have been previously observed in solvates of both squaramide and urea derivatives [19,24]. These types of 1-D arrangements are usually confined in channel-type architectures which are of great interest due the possibility to remove the solvent, generating porous materials for several applications [25].

Along the same direction (101), each symmetrically independent **A** units (1 and 2) also forms infinite 1-D chains (Figure 7a). These are both built via set of C-H∙∙∙O interactions (H∙∙∙O distances are 2.39 Å and 1.95 Å for molecule 1 and 2.39 Å and 2.20 Å for molecule 2), involving the methyl groups of the complexed DMSO molecule and the C=O groups of an adjacent **H_2_L** molecule. This kind of 1-D chains results from the squaramide function that bear both H-bond donors and acceptors and represent a robust feature in DMSO solvate of squaramide derivatives. [10,18,26,27]. Along the (10$\bar{1}$) direction (Figure 7b) of the unit cell, the crystal packing develops alternating the two types of independent 1-D chains and DMSO chains, connected each other via set of C-H∙∙∙O weak interactions (H∙∙∙O distances are in the range 2.31Å and 2.48 Å). Along the (010) direction (Figure 7c), each independent chain is related to an adjacent one by a *2_1_* screw axis, interacting with chains of the same type via π∙∙∙π interactions (inter-planar distance 3.42 Å) and resulting in the channel-type pattern shown in Figure 7c. 

**Figure 8:**
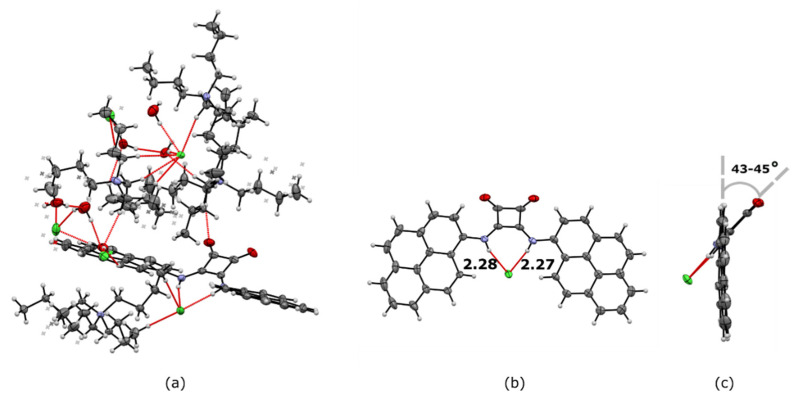
Crystal structure of **H_2_L**∙Cl^−^∙TBA^+^∙3(TBA + Cl^−^) ∙ 7.5 H_2_O (**B**). (**a**) Asymmetric unit of **B**; (**b**) receptor unit and main host-guest intermolecular interactions viewed along a direction perpendicular to the plane of pyrene rings; (**c**) receptor unit viewed along the plane of pyrene rings. The range of values of interplanar angles between the squaramide cyclobutene plane and the pyrene planes is also showed. Thermal ellipsoids drawn at the 50% probability level, disordered atoms drawn using a “wireframe style”.

Single crystals of the compound **H_2_L**∙Cl^−^∙TBA^+^ ∙3(TBA^+^Cl^−^)∙7.5 H_2_O (**B**) were obtained from THF, by slow evaporation of a solution of the receptor in the presence of an excess of TBACl (Figure 8). From this sample, yellow lath-shaped crystals suitable of single crystal X-ray diffraction were isolated and analyzed. The adduct crystallized in the triclinic crystal system (space group *P*$\bar{1}$), with a single ligand molecule, four chloride ions, four TBA ions and seven and half solvent water molecules in the asymmetric unit (Z’ = 1). The structure contains some disordered, principally affecting some of the aliphatic branches of TBA^+^ counter ions and some interchange of the Cl^−^ and H_2_O positions (see SM for further details). 

**Figure 9:**
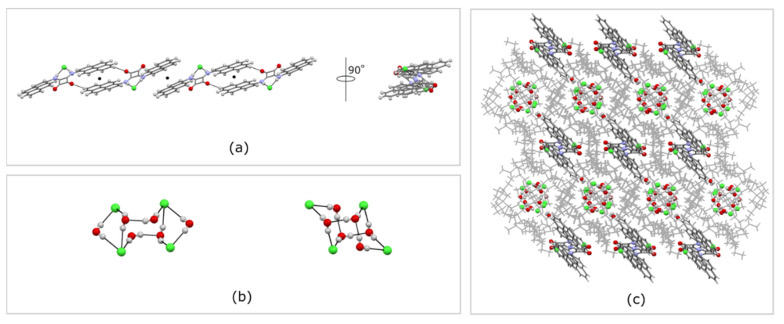
Crystal structure of **B**. (**a**) Centro-symmetric dimers view along two perpendicular directions of **B**; (**b**) H-bonded Cl^−^—H_2_O oligomeric clusters; (**c**) Crystal packing of **B** viewed down the (110) direction. Inversion symmetry indicated as black circles, TBA^+^ molecules indicated in a “wireframe style” in grey.

The receptor **H_2_L** in **B** shows a non-planar conformation similar to that observed in the case of **A**, only differing for the value of the angle between the plane of the cyclobutene spacer and the plane of the pyrene pendant arms, that in this case is in the range 43–45˚ (see Figure 8c). The pseudo-cavity is populated by one of the symmetrically independent Cl^−^ anions that interacts via N-H∙∙∙Cl^−^ with the two N-H donors (H∙∙∙Cl distance 2.28(2) Å and 2.27(3) Å). This is consistent with the solution studies that confirmed an affinity of **H_2_L** towards Cl^−^. The receptor **H_2_L** interacts with an adjacent molecule to form a H-bonded centro-symmetric dimer (Figure 9a), connected via weak C-H∙∙∙O interactions (C∙∙∙H distance is 2.42 Å), with the pyrene rings of the two adjacent molecules stacked each other and presumably interacting via π∙∙∙π interactions (inter-planar distance 3.50 Å). These develop along the (110) direction, related by inversion symmetry and surrounded by TBA^+^ cations that form a channel-like pattern. These are packed along the remaining two directions alternating clusters of H-bonded Cl^−—^H_2_O clusters (H∙∙∙O distances are in the range 1.91^−^1.95 Å and H∙∙∙Cl^−^ distances are in the range 2.13^−^2.75 Å), each surrounded by TBA^+^ cations.

The analysis of the crystal structures clearly suggests that **H_2_L** has affinity for both DMSO and Cl^−^ species. This is consistent with the fact that single crystals of B could be obtained only from THF. This competition might have relevance in view of the results of the anion binding in the liquid state. ^1^H-NMR titrations have been performed using DMSO-*d_6_*/0.5% water as a solvent mixture. As the consequence, the solvent and Cl^−^ anions compete for the interactions with the receptor unit. This might explain the small association constant calculated for the 1:1 **H_2_L**-Cl^−^ adduct. 

### 2.5. DFT Studies

**Figure 10:**
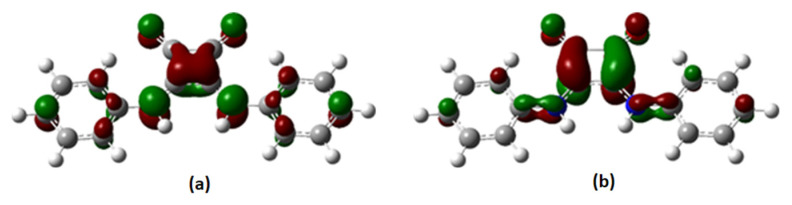
KS-HOMO (**a**) and KS-LUMO (**b**) calculated for **H_2_L’**. Cutoff value = 0.05 |e|.

In order to better understand the structural features of **H_2_L**, we performed DFT calculations, which have proved an invaluable tool in understanding a large variety of systems, including catalysts [28], NLO dyes [29], metal drugs [30,31], and sensors [32,33]. In this context, theoretical calculations carried out at the DFT level may not only explain the spectroscopic and electrochemical features of systems structurally characterized, but they can also give useful hints in understanding discrete or extended chemical systems not fully characterized yet. Following the previous work by Taylor and coworkers, [19] the diphenylsquaramide **H_2_L’** was adopted as a simplified model compound. The metric parameters optimized for **H_2_L’** are in very good agreement with the relevant structural parameters previously reported [34]. In perfect agreement with the previous theoretical results, **H_2_L’** is optimized as a planar structure, unless other interactions (such as the dimer formation) are taken into account. An analysis of the occupied frontier molecular orbitals shows that the Kohn-Sham (KS) HOMO is a π*MO built up of the *2p_z_* atomic orbitals (AOs) in Figure 10. These are perpendicular to the squarate ring plane, while the KS-LUMO is π*-MO, antibonding with respect to the (O)C–C(O) and (N)C–C(N) bonds, with minor contributions from the 2p_z_ AOs of the oxygen and nitrogen atoms.

As discussed above, ^1^H-NMR experiments clearly point out for the interaction of **H_2_L** with the Cl^–^ anion in DMSO/0.5% H_2_O solution. The **H_2_L’**·Cl^–^ adduct was therefore optimized, showing the chloride anion hydrogen-bonded symmetrically by both N–H groups of the squaramide, in agreement with the crystal structure refined for **B**. This interaction lowers the natural charge [35,36] on the chloride (Q_Cl_ = −0.808 |e|) by two Cl→H–N donations quantified in 31.12 kcal mol^–1^ each by means of a second order perturbation theory (SOPT) analysis of the Fock matrix in NBO basis (Cl···H = 2.086 Å; H···Cl···H 62.98°). With the aim to evaluate the adduct stability in a DMSO solution, solvation was kept into account at IEF-PCM SCRF level. The metric parameters optimized in DMSO solution were found to be very close to those calculated in the gas phase (Cl···H = 2.170 Å; H···Cl···H 61.43°), while the Cl→H–N interaction **H_2_L’**·Cl^–^ was only marginally weakened (23.33 kcal mol^−^^1^).

We also investigated the mono- and deprotonated forms **HL’^−^** and **L’^2−^**. There is preservation of the squaramide core upon deprotonation with only minor variations in the relevant bond lengths. The KS-HOMO and KS-LUMO of the deprotonated anion **L’**^2–^ are qualitatively analogue to that of **H_2_L’**, the HOMO-LUMO energy gap passing from 4.460 to 3.447 eV for **H_2_L** and **L^2^**^−^, respectively. An analysis of the natural charge distribution shows that the negative charge of the dianion is almost entirely concentrated on the N and O atoms (Q_O_ = −0.727 and Q_N_ = −0.659 |e|, respectively). Both the frontier MO composition and the charge distribution analysis clearly indicate that both the (C)O and the N^–^ sites can act as donors towards metal ions. 

**Figure 11:**
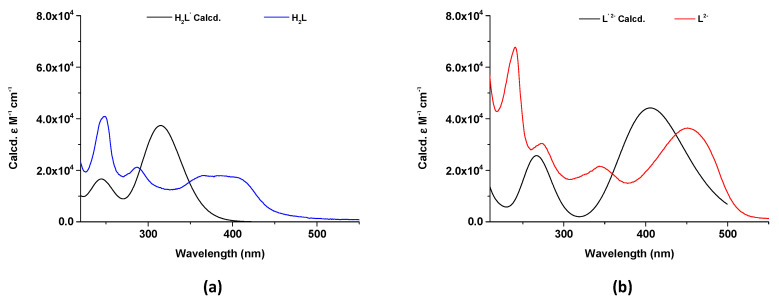
(**a**) Overlapping of TD-DFT simulated UV-Vis absorption spectra for **H_2_L’** (black line) with the UV-Vis absorption spectrum of **H_2_L** (blue line); (**b**) Overlapping of TD-DFT simulated UV-Vis absorption spectra for **L’**^2–^ (black line) with the UV-Vis absorption spectrum of **L^2^**^−^ (red line).

Time-dependent DFT (TD-DFT) calculations were carried out on **H_2_L’** and **L’**^2**−**^. Largerly in both cases, the lowest-energy allowed transition is attributed (100% and 73% for **H_2_L** and **L^2^**^−^, respectively) to a π–π* HOMO-LUMO one-electron vertical excitation involving the squarate ring (Figure 10). Under deprotonation, the transition, calculated at 3.918 eV (*f* = 0.823) for **H_2_L’** (3.729 eV, *f* = 0.975 in DMSO), undergoes a bathochromic shift and it is calculated to fall at 2.955 eV (*f* = 0.482) for **L’**^2–^ (3.137 eV, *f* = 0.946 in DMSO). Figure 11a,b depicts the simulated UV-Vis absorption spectra simulated for **H_2_L’** and **L’**^2**−**^ based on TD-DFT calculations in comparison with **H_2_L** and its deprotonated form **L^2^**^−^.

A comparison with the experimental UV-Vis absorption spectra (Figure 11a,b and Figures S2 and S7 in SM) shows a very good qualitative agreement between TD-DFT calculated data and spectroscopic measurements. This supports the hypothesis that the absorption bands at 411 nm and 465 nm in DMSO/0.5% water and at 365 and 450 nm in MeCN are related to the fully protonated and deprotonated forms of **H_2_L**.

## 3. Materials and Methods 

All starting materials and solvents were purchased from commercial sources TCI (Tokio, Japan) and Aldrich (Darmstadt, Germany) and, when necessary, the solvents were distilled and dried according to standard literature techniques. All reactions were performed in oven-dried glassware under a slight positive pressure of nitrogen. Melting point measurements were determined in capillaries, using melting point apparatus BUCHI M-560 (30–240 °C, Flawil, Switzerland). ^1^H-NMR and ^13^C NMR spectra were determined on a Bruker Avance 600 MHz (Nicholas Terrace, New York, NY, USA). Chemical shifts for ^1^H-NMR are reported in parts per million (ppm), calibrated to the residual solvent peak set, with coupling constants reported in Hertz (Hz). The following abbreviations were used for spin multiplicity: s = singlet, d = doublet, t = triplet, m = multiplet. Chemical shifts for ^13^C NMR are reported in ppm, relative to the central line of a septet at δ = 39.52 ppm for deuteriodimethylsulfoxide. Infrared (IR) spectra were recorded on a NICOLET 5700 FT-IR spectrophotometer and reported in wavenumbers (cm^−^^1^). Elemental analyses were obtained using a PerkinElmer Series II-2400 (Waltham, MA, USA). Absorption spectra were recorded on a Thermo Nicolet Evolution 300 spectrophotometer (Madison, WI, USA). Fluorescence spectra were recorded on a Cary Eclypse spectrofluorimeter. Mass spectra in positive-ion mode were recorded on a triple quadruple QqQ Varian 310-MS mass spectrometer using the atmospheric-pressure ESI technique (Lancashire, England). The 20 µL of sample of binder in DMSO solution were introduced into the ESI source by a Varian HPLC pump without column, at a flow rate of 250 µL/min using a CH_3_OH:H_2_O 1:1 mixture. A dwell time of 4 s was used, needlevoltage of 4000 V, shield voltage of 600 V, housing temperature of 60 °C, drying gas temperature of 400 °C, nebuliser gas pressure of 46 PSI, drying gas pressure of 35 PSI and a detector voltage of 1490 V were used. Mass spectra were acquired in the 250–500amu range. 

### 3.1. ^1^H-NMR Titrations

Proton NMR titrations were performed by adding aliquots of the putative anionic guest (as the TBA salt, 0.075 M) in a solution of the receptor (0.005M) in DMSO-*d*_6_/0.5% water to a solution of the receptor (0.005 M). 

### 3.2. Crystallization Methods

Several attempts of crystallization have been performed in a wide range of solvents (MeCN, MeOH, EtOH, CHCl_3_, DCM, EtOAc, Hexane, THF, DMSO) by means of different methods (slow evaporation of the solvent, diffusion). **H_2_L** resulted insoluble in most of the solvents used. Indeed, we were able to dissolve **H_2_L** only in DMSO and then to collect single crystals for the solvent coordinated form of the free receptor **H_2_L ∙ 2 DMSO**. In order to collect the crystal adducts of **H_2_L** with anion, we performed crystallization in the presence of an excess of anion species. Particularly, the presence of the guest allowed to increase the **H_2_L** solubility in some of the solvents used, like THF in which a single crystal for the adduct receptor-anion in the presence of Cl^−^ was collected. Despite many attempts we were not able to obtain crystals suitable for single crystal X-ray diffraction analysis for the 2:1 Cu^2+^: **H_2_L** complex. **H_2_L** is highly insoluble in MeCN. In the attempt to characterize the complex we tried to react **H_2_L** and Cu(ClO_4_)_2_ hydrate in a 1:2 ratio in MeCN at a concentration of 1.0 × 10^−4^ M. We obtained a fluorescent turbid solution suggesting the formation of the complex. However, the concentration of the complex in solution was too low to be analyzed by ESI-MS. Moreover, the scarce solubility of **H_2_L** hampered the isolation of the complex as a solid. 

#### X-ray Data Collection

(**A**) A small portion of this sample was suspended in perfluoroether oil; a suitable yellow lath-shaped crystal (0.145 × 0.045 × 0.020 mm^3^) was selected and mounted on a MITIGEN holder with perfluoroether oil then aligned upon a Rigaku FRE+ diffractometer, equipped with VHF Varimax confocal mirrors and an AFC12 goniometer and HyPix 6000 detector. The crystal was kept at a steady T = 100(2) K during data collection, using an Oxford Cryosystems cold device. The structure was solved with the ShelXD [37] structure solution program using the Dual Space solution method and by using Olex2 [38] as the graphical interface. The model was refined with version 2018/3 of ShelXL [39] using Least Squares minimisation.

(**B**) A small portion of this sample was suspended in perfluoroether oil; a yellow (cut) lath-shaped crystal (0.198 × 0.047 × 0.030 mm^3^) was selected and mounted on a MITIGEN holder with perfluoroether oil then aligned upon a Rigaku FRE+ diffractometer, equipped with VHF Varimax confocal mirrors and an AFC12 goniometer and HyPix 6000 detector. The crystal was kept at a steady T = 100(2) K during data collection, using an Oxford Cryosystems cold device. The structure was solved with the ShelXT 2018/2 [40] solution program using dual methods and by using Olex2 [38] as the graphical interface. The model was refined with ShelXL 2018/3 [39] using least squares minimisation.

### 3.3. Theoretical Calculations

Quantum-mechanical calculations were carried out at density functional theory (DFT) [41,42] level with the Gaussian16 (rev. B.01) [41] commercial suite of computational software. For all compounds, the mPW1PW functional [43] was paralleled by the def2-SVPD [44,45] basis set for all atomic species. Solvation was implicitly taken into account by means of the polarizable continuum model in its integral equation formalism (IEF-PCM) [46], describing the cavity of the compounds within the reaction field (SCRF) through a set of overlapping spheres. The nature of the energy minima at the optimized geometries were verified by a vibrational analysis, computed by determining the second derivatives of the energy with respect to the orthogonal Cartesian atomic coordinates and subsequently transforming to mass-weighted coordinates. Natural Bonding Orbitals, natural charges, and Wiberg bond indices were calculated at the optimized geometries. A Second Order Perturbation Theory (SOPT) Analysis of Fock Matrix in NBO Basis was also carried out to investigate intramolecular donor–acceptor and hydrogen bonding interactions. TD-DFT calculations (100 states) were carried out at the optimized geometries. The programs Chemissian, Molden 6.6 [47], and GaussView 6 were used to analyze optimized geometries, the KS-MO composition and natural charge distributions, and to simulate UV-Vis absorption spectra based on TD-DFT data.

### 3.4. Synthesis of **H_2_L**

To a stirred solution of 3,4-diethoxycyclobut-3-ene-1,2-dione (0.2 g, 1.18 mmol) and zinc trifluoromethanesulfonate (20 mol %) in toluene/DMF (19:1 *v*/*v*, 4 mL) the 1-pyrene amine (0.560 g, 2.58 mmol) was added. This solution was heated at 100 °C and then stirred for 24 h. A precipitate formed as the solution cooled to room temperature, subsequently isolated by filtration. The solid was further washed with methanol (3 × 5 mL) and dried under reduced pressure to remove the residual methanol obtaining the product as crude red solid. Yield = 0.3 g, 50% M.p.: > 250 °C; ^1^H-NMR (600 MHz, DMSO-*d_6_*) δ (ppm) = 10.65 (s, 2H, -NH), 8.46 (d, *J* = 12 Hz, 2H, pyrene CH), 8.29 (t, *J* = 6 Hz, 2H, pyrene CH), 8.25 (d, *J* = 6 Hz, 2H, pyrene CH), 8.17 (m, 4H, pyrene CH), 8.09 (m, 4H, pyrene CH), 7.99 (m, 2H, pyrene CH) ^13^C NMR (150.9 MHz, DMSO-*d_6_*) δ (ppm) = 167.7, 132.0, 131.4, 130.9, 129.4, 128.7, 128.3, 128.0, 127.5, 127.0, 126.7, 125.8, 125.7, 125.4, 124.7, 124.3, 122.8, 122.2, 121.9, 121.3. IR: 3400 cm^−1^ (stretching N-H), 1780 cm^−1^ (stretching C=O). Elemental Analysis: % found (% calc. for C_36_H_20_N_2_O_2_): C 84.94 (84.36), H 4.09 (3.93), N 5.55 (5.47). LRMS (ES+): *m/z*: 513.2 [M-H]^+^

## 4. Conclusions

In conclusion, we synthesized the new symmetric fluorescent *N,N’*-bis(1-pyrenyl) squaramide (**H_2_L**) featuring pyrene groups as fluorogenic moieties. Solution studies conducted by ^1^H-NMR spectroscopy in DMSO-*d*_6_/0.5% water and UV-Vis and fluorescence spectroscopies in DMSO/0.5% water and MeCN highlighted a low affinity of **H_2_L** for Cl^−^ and deprotonation of the receptor in the presence of basic anions such as F^−^, CN^−^, and OH^−^. The low affinity towards Cl^−^ could be attributed to a competition between the DMSO and the anion for the receptor as suggested by solid state studies. Indeed, in the less competitive solvent MeCN optical spectroscopies point out a stronger interaction of **H_2_L** with Cl^−^ than DMSO. Interestingly, a selective fluorescent emission enhancement, even detectable by naked eye, was observed in the presence of copper(II) in MeCN. Indeed, the fluorescence of the receptor was switched-on as the consequence of the formation of the 2:1 Cu^2+^:**H_2_L** complex. The deprotonation of the squaramide NHs of the complex caused by basic anions induced a dramatic change in the fluorescence of the system with the formation of a lower intensity red-shifted emission band. This might open the possibility to use the 2:1 Cu^2+^:**H_2_L** complex as fluorescent chemodosimeter for basic anions such as F^−^ and CN^−^. 

## Figures and Tables

**Table 1:** Association constants (*K*_a_/M^−^^1^) for the formation of the 1:1 adducts of **H_2_L** with anions added as their tetrabutylammonium salts in DMSO-*d*_6_/0.5% water at 298 K.

	F^−^	CN^−^	BzO^−^	Cl^−^	Br^−^	I^−^
**H_2_L**	deprot	deprot	deprot	98 ± 6	<10	<10

## Data Availability

Crystallographic data have been deposited at CCCD (CIFs deposition numbers 2054755 and 2054756).

## Supplementary Materials

The following are available online, Figure S1 ^1^H NMR spectrum of H_2_L in DMSO-*d*_6_/0.5% water at 298 K; Figure S2: ^13^C NMR spectrum of H_2_L in DMSO-*d*_6_/0.5% water at 298 K; Figure S3. (a) ^1^H NMR Stack plot of H_2_L in DMSO-*d*_6_/0.5% water at 298 K in the presence of increasing molar ratios of TBABzO; (b) colour change of a solution of H_2_L (5 × 10^−3^ M) upon the addition of a solution of TBABzO (7.5 × 10^−2^ M) due to the H_2_L deprotonation; Figure S4. ^1^H NMR titration of H_2_L (0.005 M) the presence of increasing molar ratios of TBACl (0.075 M) in DMSO-*d*_6_/0.5% water at 298 K; Figure S5. ^1^H NMR Stack-plot of H_2_L (0.005 M) in the presence of increasing molar ratios of TBABr (0.075 M) in DMSO-*d*_6_/0.5% water at 298 K; Figure S6. ^1^H NMR Stack plot of H_2_L (0.005 M) in the presence of increasing molar ratios of TBAI (0.075 M) in DMSO-*d*_6_/0.5% water at 298 K; Figure S7. UV−Vis titration of H_2_L (2.1 × 10^−5^ M) with an increasing amount of (a) TBAOH; (b) TBACN; (c) TBAF; (d) TBABzO; and (e) TBACl in DMSO/0.5% water; Figure S8. UV−Vis titration of H_2_L (1.0 × 10^−5^ M) with an increasing amount of (a) TBAOH; (b) TBACN; (c) TBAF; (d) TBABzO; and (e) TBACl in MeCN; Figure S9. Spectrofluorimetric titrations of H_2_L (1.0 × 10^−5^M) with an increasing amount of (a) TBAOH; (b) TBACN; (c) TBAF; (d) TBABzO; and (e) TBACl in MeCN, λ_exc_ = 350 nm; Figure S10: UV−Vis titration of H_2_L (1.0 × 10^−5^ M) with an increasing amount of (a) Cd^2+^; (b) Cu^2+^; (c) Hg^2+^; (d) Ni^2+^; (e) Pb^2+^; and (f) Zn^2+^ in MeCN. Figure S11. Spectrofluorimetric studies of the H_2_L copper-complex (H_2_L: Cu^2+^ 1:2) in MeCN (λ_exc_ = 350 nm) in the presence of increasing amount of (a) TBACN; (b) TBAF; Figure S12: UV-Vis titrations of the H_2_L copper-complex (H_2_L: Cu^2+^ 1:2) in MeCN with increasing amount of (a) TBAOH; (b) TBACN; (c) TBAF; (d) TBACl; Figure S13. UV-Vis titration data of H_2_L (1.0 × 10^−5^ M) upon the addition of increasing amount of Cu(ClO_4_)_2_ hydrate (2.5 × 10^−3^ M) in MeCN; Table S1. Crystallographic parameters for crystal structures A and B; Table S2. Hydrogen Bond information for A and B.

## Sample Availability

Samples of the compounds are available from the authors.
