# Supplementary material for: Switching-On Fluorescence by Copper (II) and Basic Anions: A Case Study with a Pyrene-Functionalized Squaramide
Source: Molecules. 2021 Feb 28;26(5):1301. doi: 10.3390/molecules26051301 (PMC7957675; doi:10.3390/molecules26051301)
Supplement: Supplementary file 1 [file molecules-26-01301-s001.pdf]

Switching-on Fluorescence by Copper (II) and Basic Anions: a Case Study with a Pyrene-Functionalised Squaramide

Giacomo Picci, Jessica Milia, Maria Carla Aragoni, Massimiliano Arca, Simon J. Coles, Alessandra Garau, Vito Lippolis [1], Riccardo Montis [2], James B. Orton, and Claudia Caltagirone.

Supplementary Information

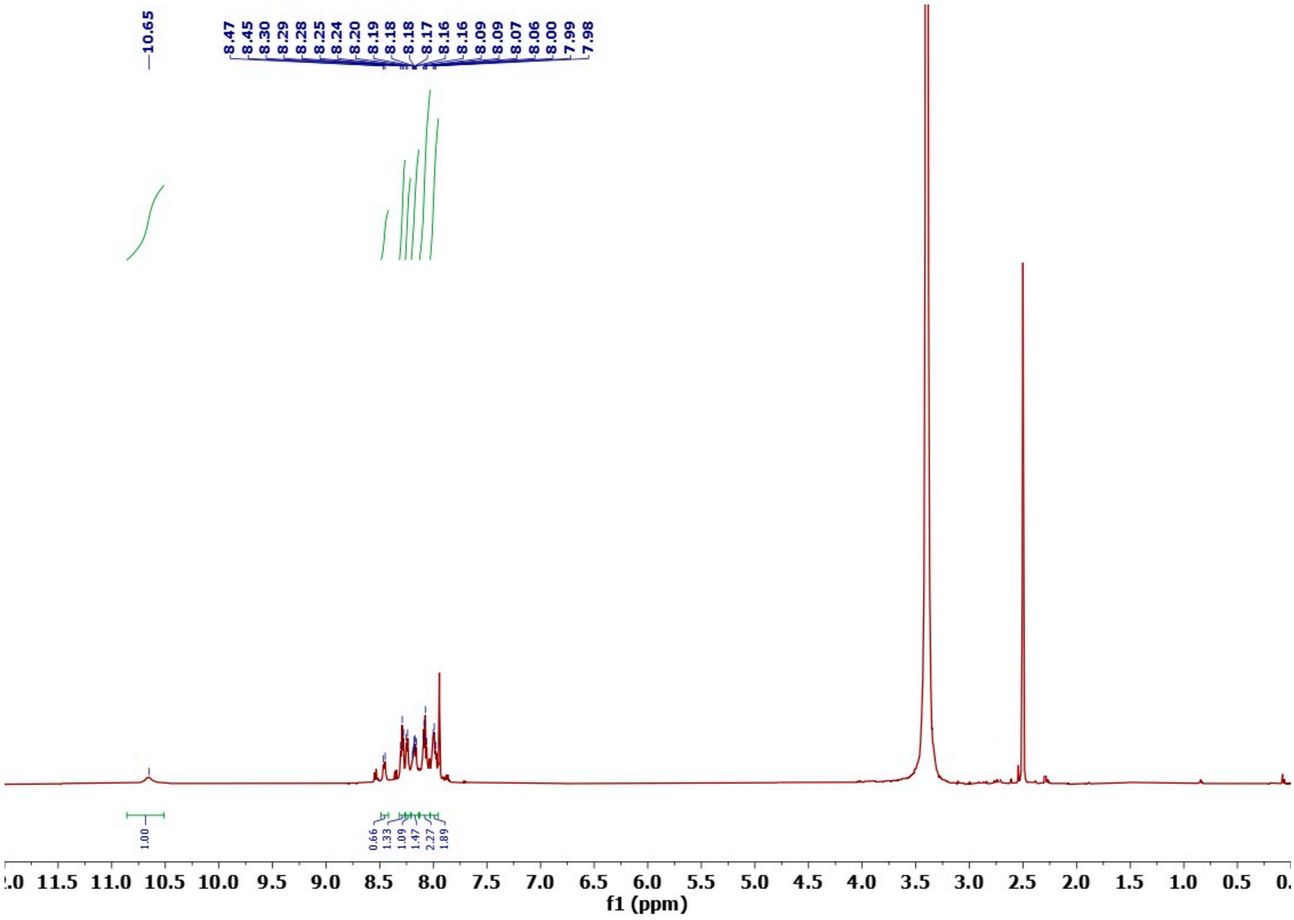

Figure S1 ^{1}H NMR spectrum of $\mathbf{H_2L}$ in DMSO-d_6/0.5% water at 298 K.

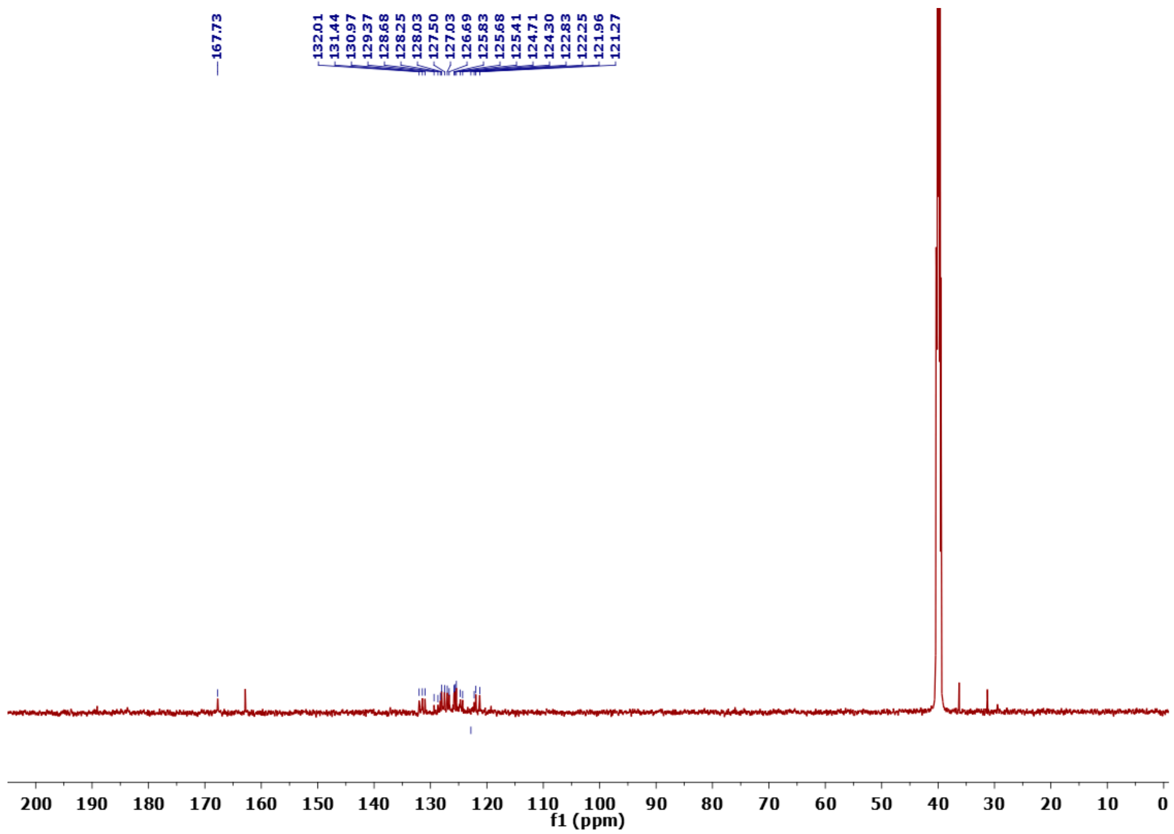

Figure S2: ^{13}C NMR spectrum of **H_2L** in DMSO-d_6/0.5% water at 298 K.

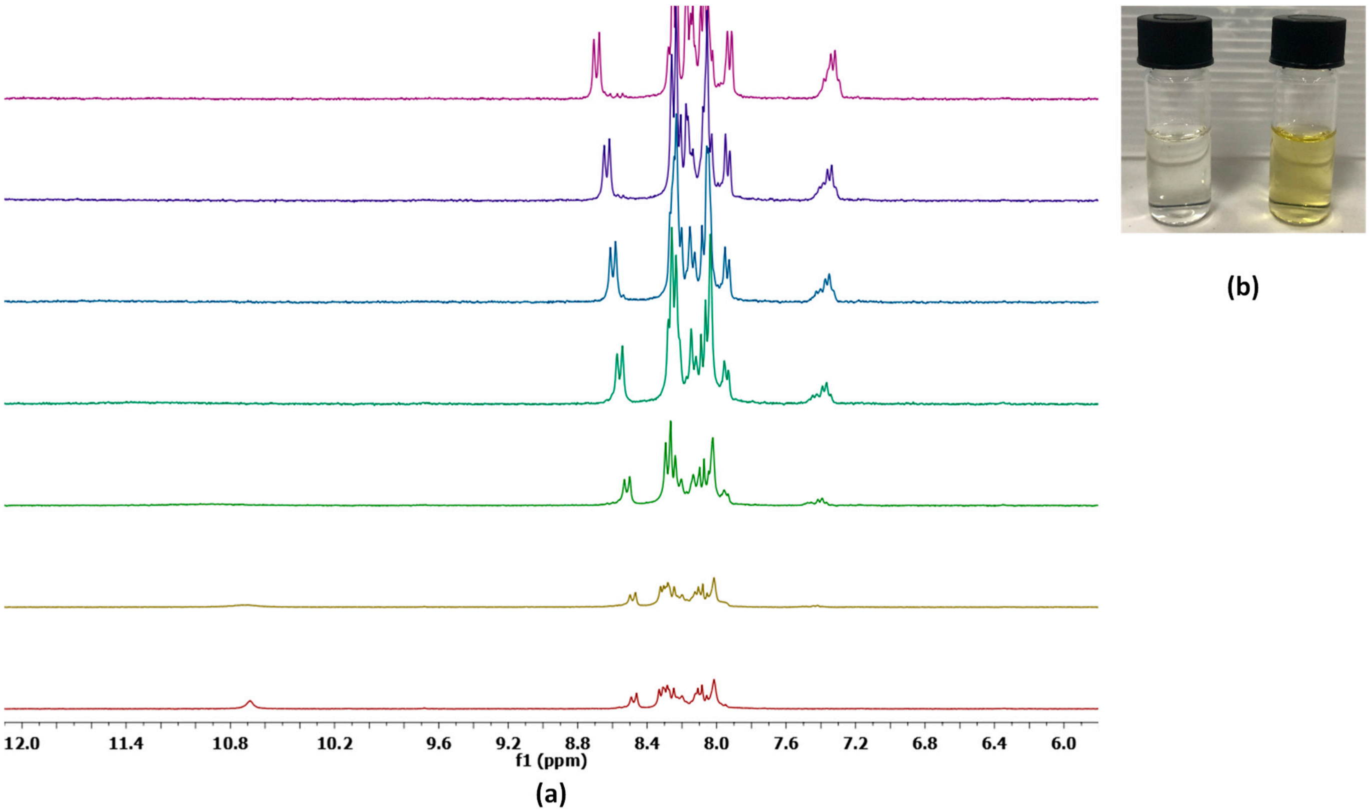

Figure 3. (**a**) ^{1}H NMR Stack plot of $\mathbf{H_2L}$ in DMSO-d_6/0.5% water at 298 K in the presence of increasing molar ratios of TBABzO; (**b**) colour change of a solution of $\mathbf{H_2L}$ (5×10^{-3} M) upon the addition of a solution of TBABzO (7.5×10^{-2} M) due to the $\mathbf{H_2L}$ deprotonation

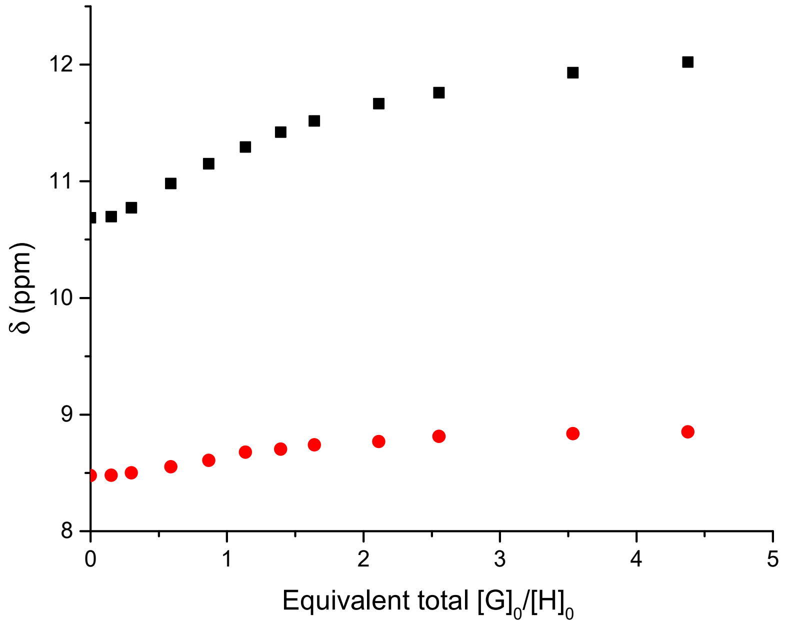

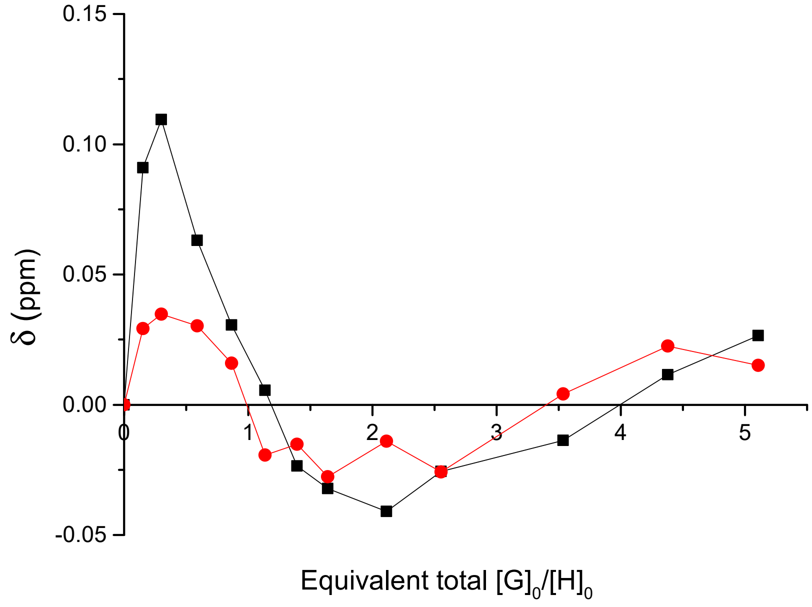

K	K error (%)	SSR	Datapoints fitted	Params fitted	H coeffs	HG coeffs	Raw coeffs 1	Raw coeffs 2
98,0825653	6,38908176	0,03633805	26	3	10,687	12,7578989	10,687	12,7578989
					8,4774	9,08851127	8,4774	9,08851127

Figure S4. ^{1}H NMR titration of **H_2L** (0.005 M) in the presence of increasing molar ratios of TBACl (0.075 M) in DMSO-d_6/0.5% water at 298 K.

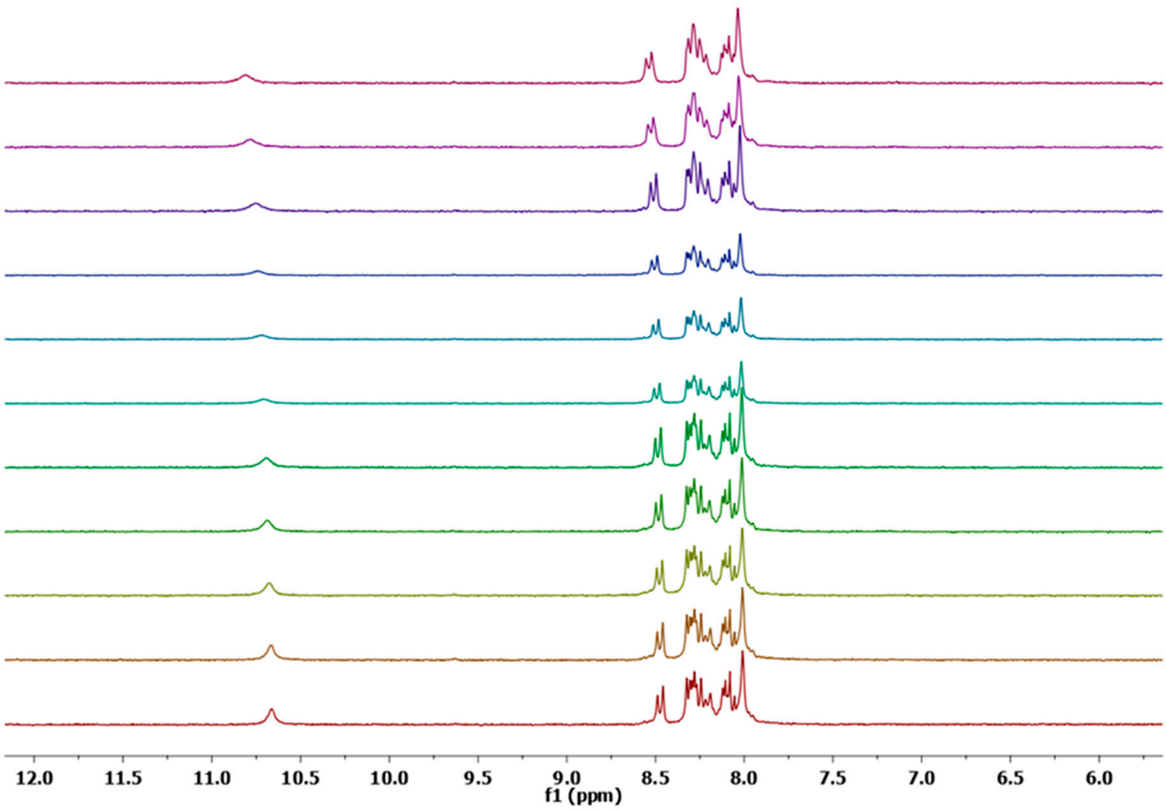

Figure S5. ^{1}H NMR Stack-plot of **H$_2$L** (0.005 M) in the presence of increasing molar ratios of TBABr (0.075 M) in DMSO-d_6/0.5% water at 298 K.

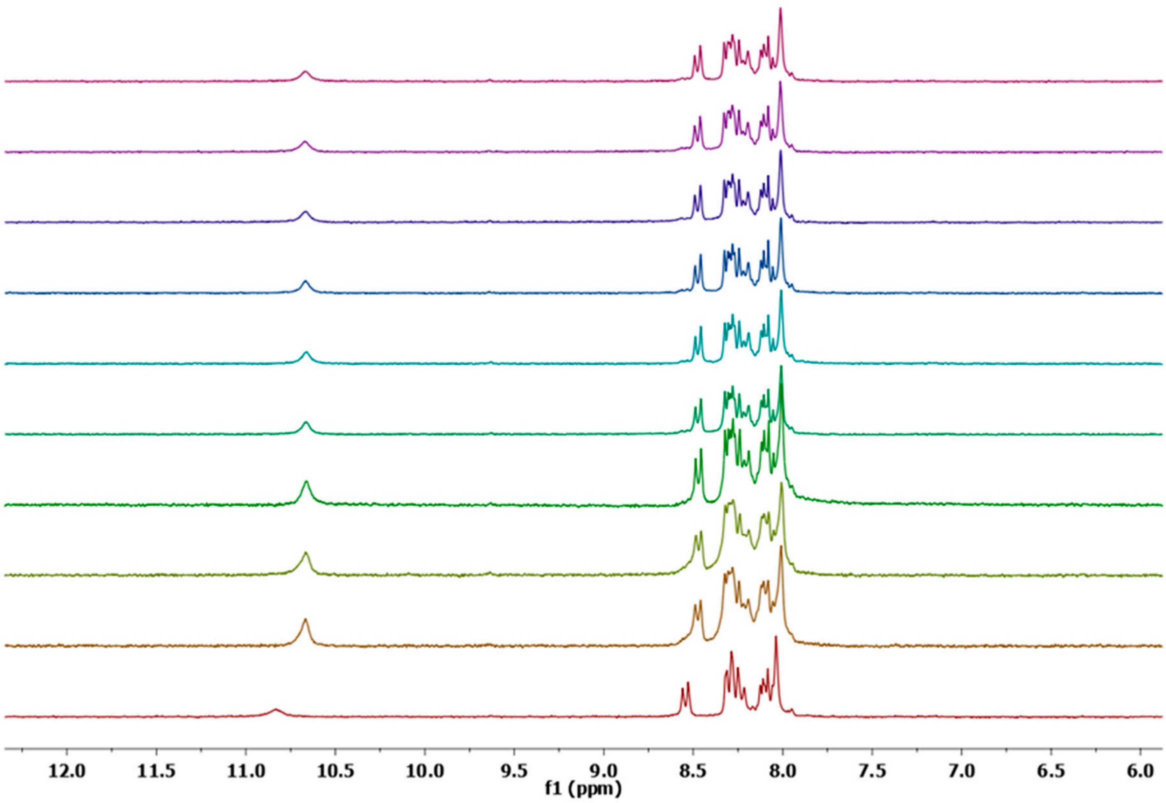

Figure S6. ^{1}H NMR Stack plot of **H_2L** (0.005 M) in the presence of increasing molar ratios of TBAI (0.075 M) in DMSO-d_6/0.5% water at 298 K.

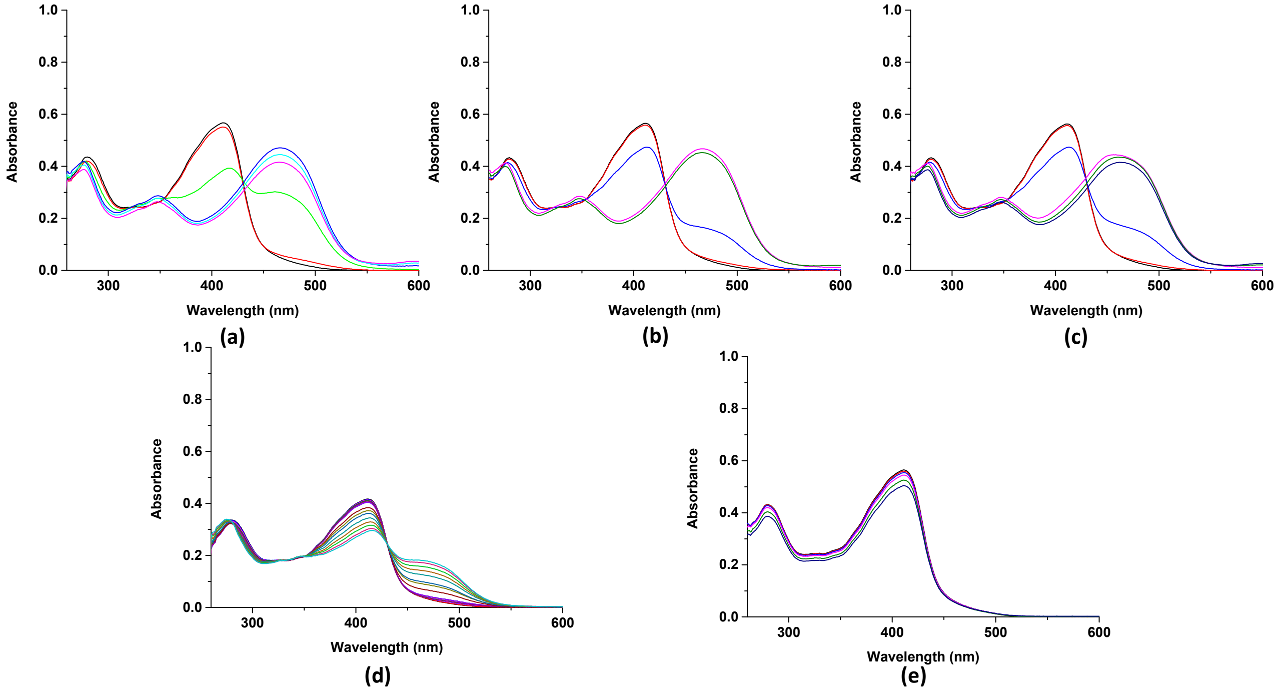

Figure S7. UV−Vis titration of **H_2L** (2.1×10^{-5}M) with an increasing amount of (**a**) TBAOH; (**b**) TBACN; (**c**) TBAF; (**d**) TBABzO; and (**e**) TBACl in DMSO/0.5% water.

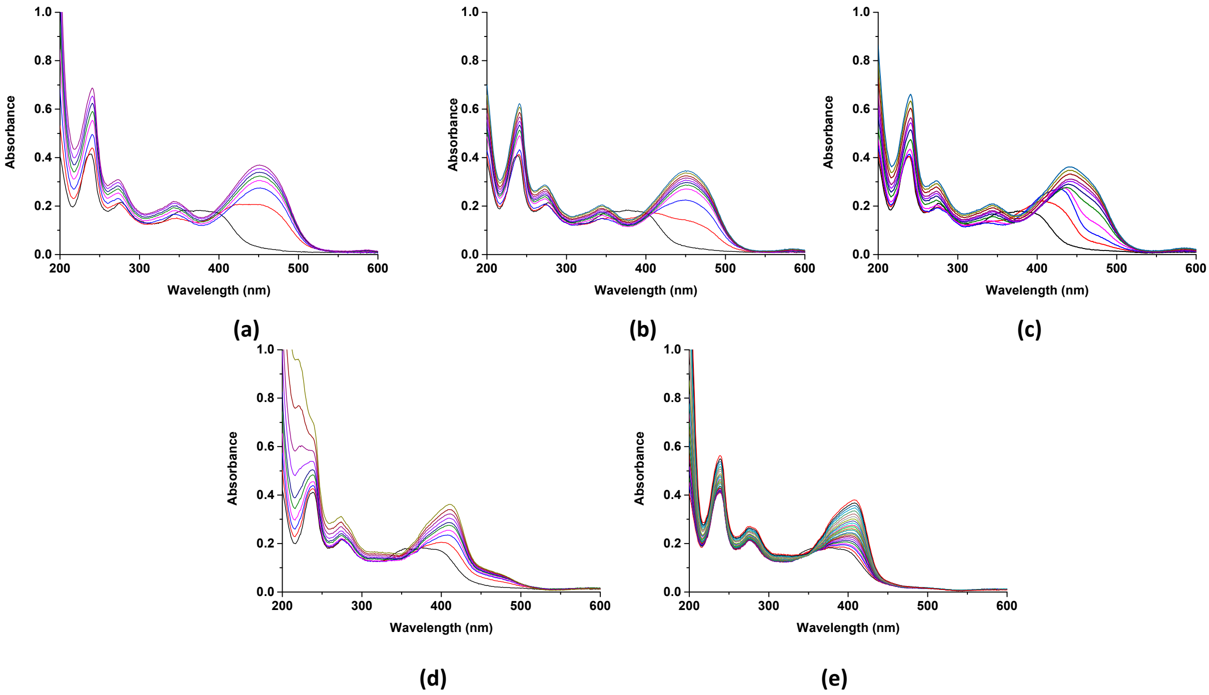

Figure S8. UV−Vis titration of H_2L (1.0 x 10^{-5}M) with an increasing amount of (**a**) TBAOH; (**b**) TBACN; (**c**) TBAF; (**d**) TBABzO; and (**e**) TBACl in MeCN.

.

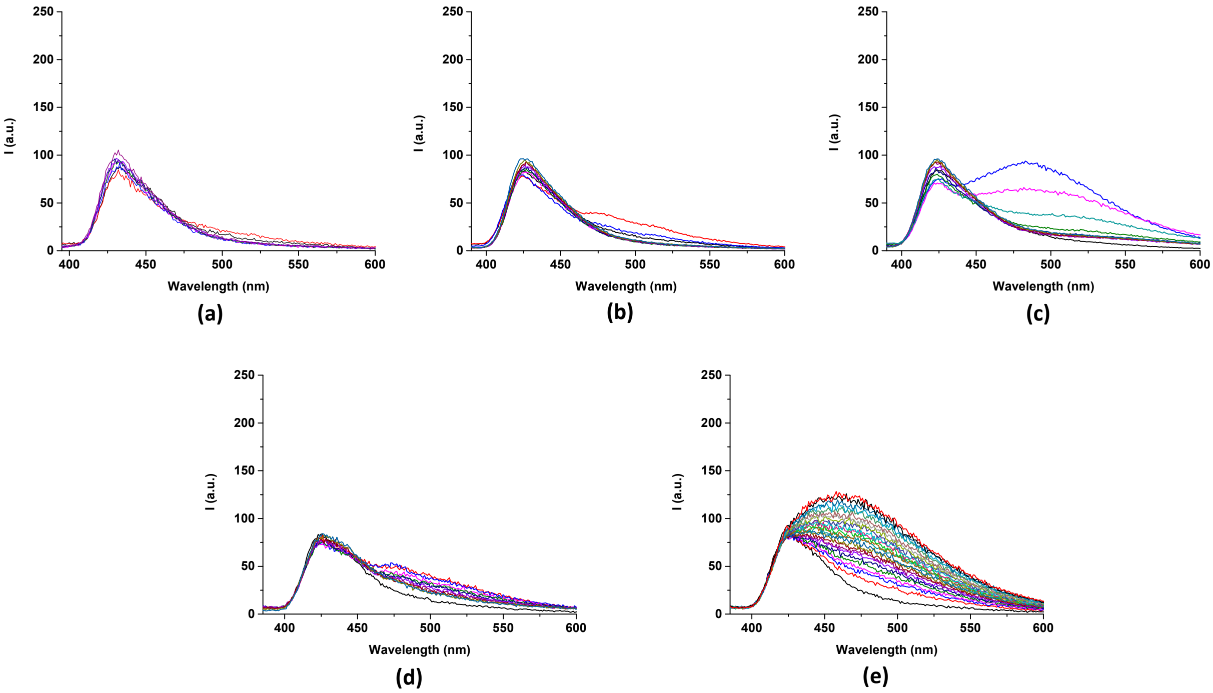

Figure S9. Spectrofluorimetric titrations of H_2L (1.0 x 10^{-5}M) with an increasing amount of (**a**) TBAOH; (**b**) TBACN; (**c**) TBAF; (**d**) TBABzO; and (**e**) TBACl in MeCN, λ_{exc} = 350 nm.

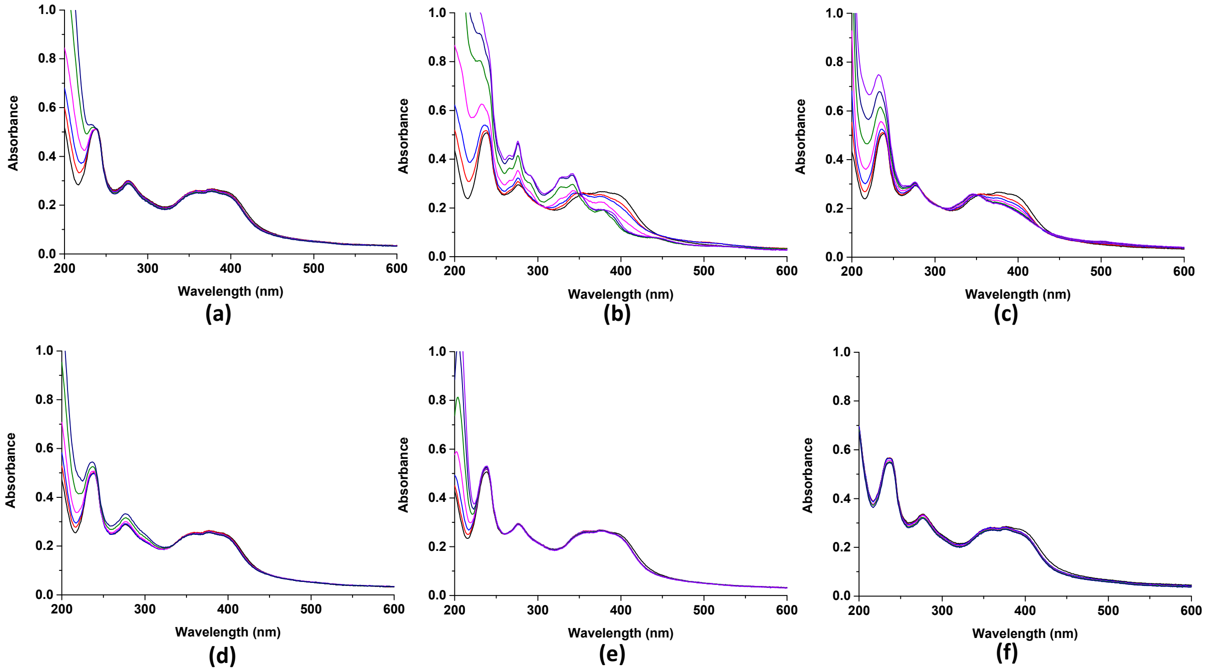

Figure S10: UV−Vis titration of H_2L (1.0 x 10^{-5}M) with an increasing amount of (**a**) Cd^{2+}; (**b**) Cu^{2+}; (**c**) Hg^{2+}; (**d**) Ni^{2+}; (**e**) Pb^{2+}; and (**f**) Zn^{2+} in MeCN.

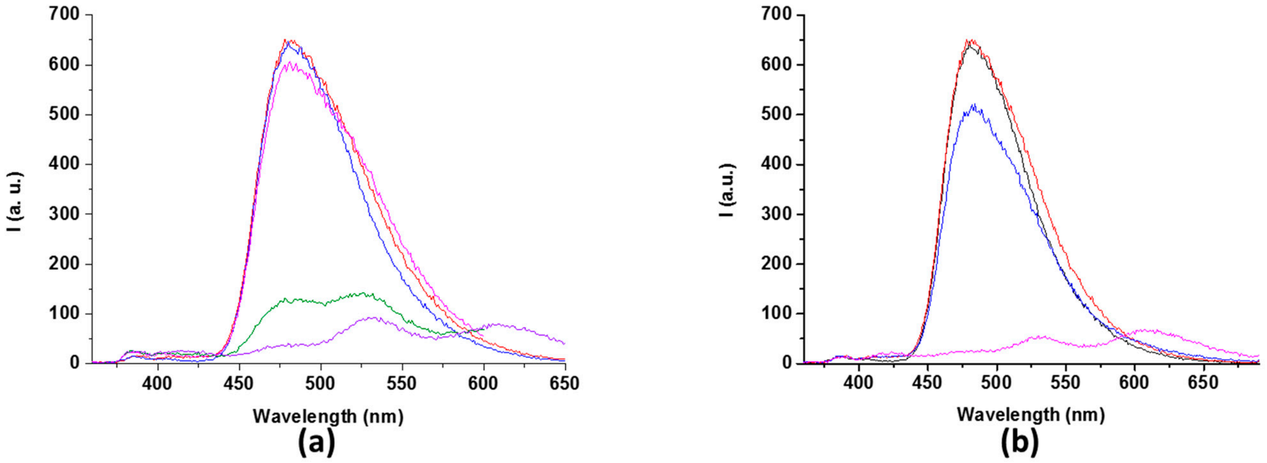

Figure S11. Spectrofluorimetric studies of the **H**$_2$**L** copper-complex (**H**$_2$**L** : Cu^{2+} 1:2) in MeCN (λ_{exc} = 350 nm) in the presence of increasing amount of (**a**) TBACN; (**b**) TBAF.

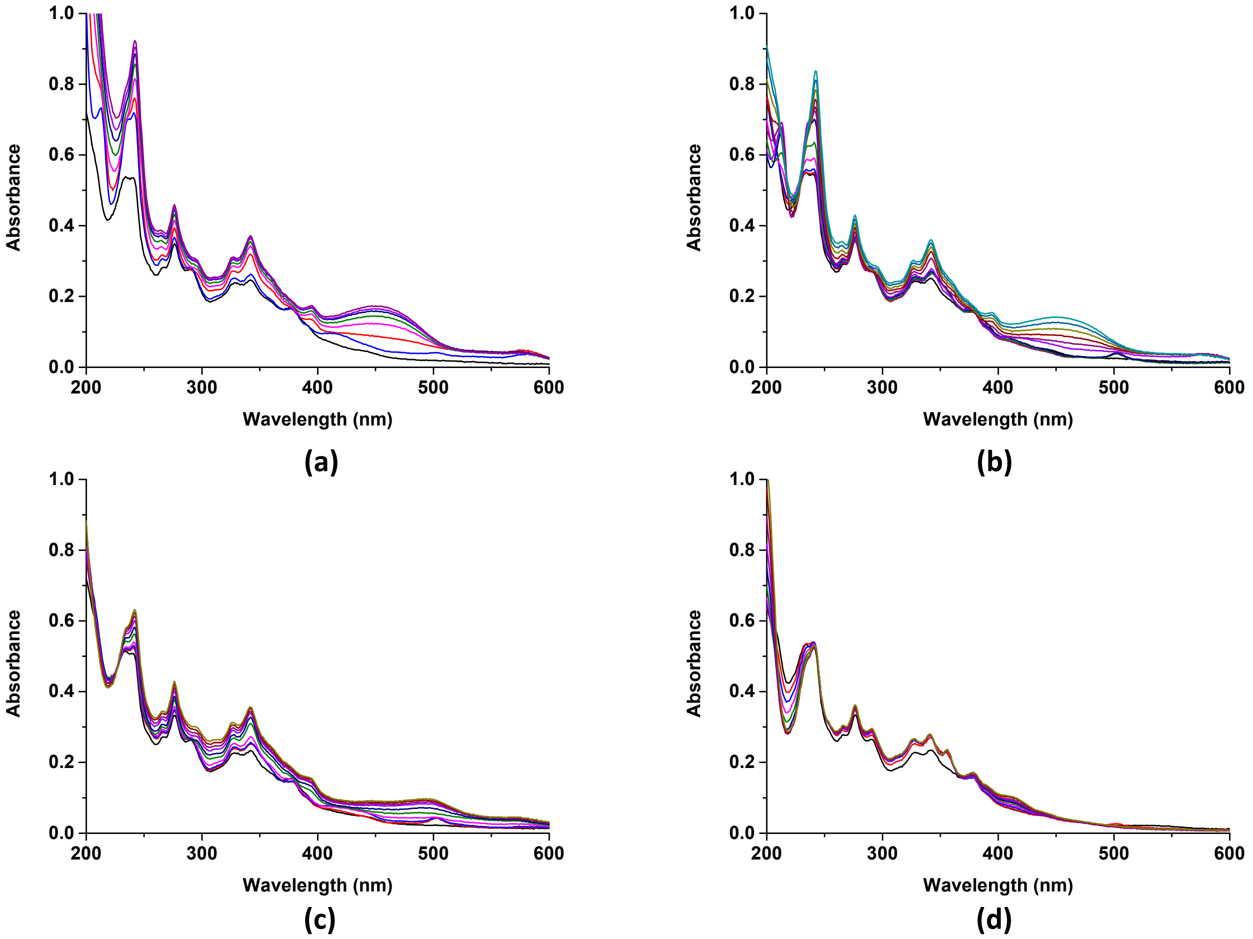

Figure S12: UV-Vis titrations of the **H**$_2$**L** copper-complex (**H**$_2$**L** : Cu^{2+} 1:2) in MeCN with increasing amount of (**a**) TBAOH; (**b**) TBACN; (**c**) TBAF; (**d**) TBACl.

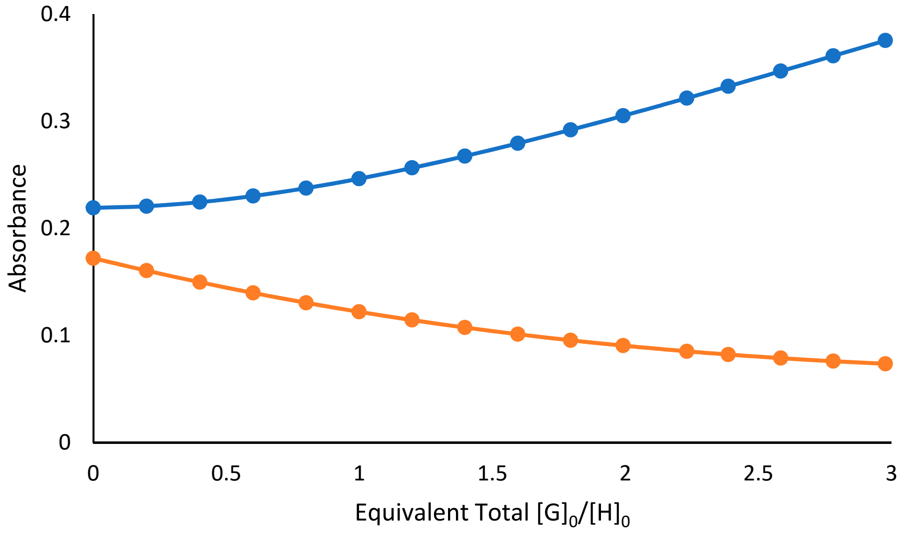

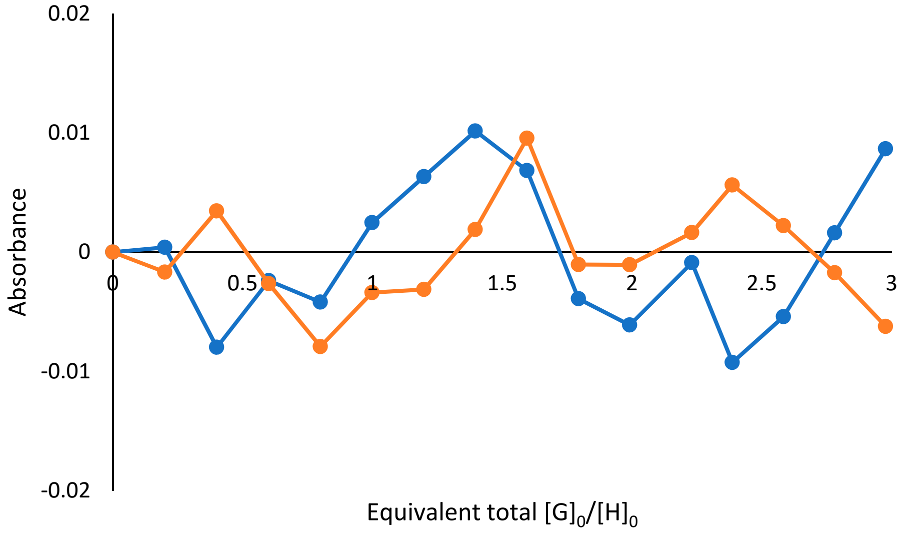

K_{11}	K_{12}	K_{11} error (%)	K_{12} error (%)	SSR	Datapoints fitted	Params fitted	H coeffs	HG coeffs	HG2 coeffs	Raw coeffs 1	Raw coeffs 2	Raw coeffs 3
33.6447536	13977402.6	4.14416623	5.63971518	0.00081176	32	6	21597.6331	508332.213	86265.0918	21597.6331	508332.213	86265.0918
							16962.5247	-17226175.6	23806.5309	16962.5247	-17226175.6	23806.5309

http://app.supramolecular.org/bindfit/view/a9c14fc1-86c2-4af4-b64e-75df163ed016

Figure S13. UV-Vis titration data of H_2L (1.0×10^{-5} M) upon the addition of increasing amount of $Cu(ClO_4)_2$ hydrate (2.5×10^{-3} M) in MeCN.

Single Crystal X-ray Diffraction

Table 1. Crystallographic parameters for crystal structures **A** and **B.**

Compound	H_2L · 2 DMSO	$H_2L{\cdot}Cl^-{\cdot}TBA^+ \cdot 3(TBA+Cl^-) \cdot 7.5H_2O$
	A	B
**CCDC Deposition N**	**2054755**	**2054756**
**Formula**	$C_{36}H_{20}N_2O_4 .2(C_2H_6SO)$	$\{[C_{35}H_{22}N_2O_2.Cl]^- 3Cl^- 4(C_{16}H_{36}N)^+ .7H_2O\}$[10]
**Dcalc./ g cm-3**	1.396	1.116
**/mm^{-1}**	0.215	0.168
**Formula Weight**	668.79	1759.28
**Color**	yellow	yellow
**Shape**	lath	(cut) lath
**Size/mm^3**	0.145×0.045×0.020	0.198×0.047×0.030
**T/K**	100(2)	100(2)
**Crystal System**	monoclinic	triclinic
**Space Group**	P21/n	P-1
**a/Å**	16.05519(19)	15.9447(3)
**b/Å**	24.7796(3)	17.8943(3)
**c/Å**	16.06983(19)	18.6302(3)
**/°**	90	88.6300(10)
**/°**	95.6334(11)	80.1610(10)
**/°**	90	89.475(2)
**V/$Å^3$**	6362.38(13)	5235.81(16)
**Z**	8	2
**Z'**	2	1
**Wavelength/Å**	0.71075	0.71075
**Radiation type**	MoK	Mo K
**min/°**	1.888	1.947
**max/°**	27.485	27.486
**Measured Refl's.**	166804	121234
**Indep't Refl's**	14580	23977
**Refl's I≥2 (I)**	13458	19252
**R_{int}**	0.0450	0.0352
**Parameters**	1060	1275
**Restraints**	391	273
**Largest Peak**	0.786	0.837
**Deepest Hole**	-0.345	-0.330
**GooF**	1.061	1.009
**wR2 (all data)**	0.1552	0.1629
**wR2**	0.1503	0.1517
**R1 (all data)**	0.0633	0.0737
**R1**	0.0583	0.0582

Table 2. Hydrogen bond information for **A** and **B.**

Compound	D	H	A	d(D-H)/Å	d(H-A)/Å	d(D-A)/Å	D-H-A/deg
	N1	H1	O5	0.84(4)	2.01(4)	2.815(3)	161(3)
	N2	H2	O5	0.89(3)	1.90(3)	2.782(3)	168(3)
A	N3	H3	O6	0.85(4)	2.02(4)	2.822(3)	157(3)
	N4	H4	O6	0.81(4)	1.99(4)	2.791(3)	169(3)
	O9	H9A	O8	0.87	1.95	2.801(2)	166.8
	O9	H9B	Cl4A1	0.87	2.58	3.427(3)	165.4
	O9	H9B	Cl4B1	0.87	2.23	3.100(3)	173.7
	N1	H1	Cl1	0.85(2)	2.27(2)	3.1097(15)	170(2)
	N2	H2	Cl1	0.90(2)	2.28(2)	3.1539(15)	164.4(19)
	O7	H7A	Cl3B	0.87	2.24	3.093(2)	166.0
	O7	H7A	O6B	0.87	2.10	2.948(3)	163.0
	O7	H7B	Cl4A	0.87	2.22	3.083(3)	171.0
B	O7	H7B	Cl4B	0.87	2.50	3.360(3)	171.1
	O3	H3B	Cl2A	0.87	2.13	2.995(6)	171.1
	O3	H3B	Cl2B	0.87	2.34	3.203(7)	172.0
	O4	H4A	O52	0.87	1.92	2.779(3)	171.0
	O4	H4B	Cl2A	0.87	2.35	3.211(4)	173.6
	O4	H4B	Cl2B	0.87	2.25	3.110(6)	170.6
	O8	H8A	O10	0.87	1.89	2.684(4)	151.1
	O8	H8B	Cl4A	0.87	2.34	3.208(3)	175.8
	O8	H8B	Cl4B	0.87	2.41	3.269(3)	167.6
	O5	H5A	O3	0.87	1.91	2.768(2)	168.0

[1]-x,1-y,-z; [2]1-x,-y,-z.

1). Structure (A): H_2L · 2 DMSO

Crystal Data. $C_{40}H_{32}N_2O_4S_2$, M_r = 668.79, monoclinic, $P2_1/n$ (No. 14), a = 16.05519(19) Å, b = 24.7796(3) Å, c = 16.06983(19) Å, = 95.6334(11)°, = = 90°, V = 6362.38(13) Å^3, T = 100(2) K, Z = 8, Z' = 2, (MoK) = 0.215 mm^{-1}, 166804 reflections measured, 14580 unique (R_{int} = 0.0450), which were used in all calculations. The final wR_2 was 0.1552 (all data) and R_1 was 0.0583 (I > 2(I)).

A yellow lath-shaped crystal with dimensions 0.145 × 0.045 × 0.020 mm^3 was mounted on a MITIGEN holder in perfluoroether oil. Data was collected using an Rigaku FRE+ equipped with VHF Varimax confocal mirrors and an AFC12 goniometer and HyPix 6000 detector diffractometer equipped with an Oxford Cryosystems low-temperature device operating at T = 100(2) K.

Data was measured using profile data from -scans of 0.5° per frame for 33.8 s using MoK radiation. The total number of runs and images was based on the strategy calculation from the program **CrysAlisPro** (Rigaku, V1.171.40.47a, 2019). The maximum resolution achieved was = 27.485° (0.77 Å).

The diffraction pattern indexed with the total number of runs and images was based on the strategy calculation from the program **CrysAlisPro** 1.171.40.47a (Rigaku Oxford Diffraction, 2019); the unit cell was refined using 54270 reflections, 33% of the observed reflections. Data reduction, scaling, and

absorption corrections were performed using **CrysAlisPro** 1.171.40.47a (Rigaku Oxford Diffraction, 2019). The final completeness is 99.90 % (**IUCr**) out to 27.485° in .

A Gaussian absorption correction was performed using **CrysAlisPro** 1.171.40.47a (Rigaku Oxford Diffraction, 2019). Numerical absorption correction was based on Gaussian integration over a multifaceted crystal model. Empirical absorption correction used spherical harmonics as implemented in SCALE3 ABSPACK. The absorption coefficient of this material is 0.215 mm^{-1} at this wavelength (= 0.71075 Å) and the minimum and maximum transmissions are 0.732 and 1.000.

The structure solved and the space group $P2_1/n$ (# 14) determined by the ShelXD (Sheldrick, 2008) structure solution program using Dual Space and refined by Least Squares using version 2018/3 of **ShelXL** (Sheldrick, 2015). All non-hydrogen atoms were refined anisotropically. The positions of the N-H atoms H1, H2, H3, and H4 were located from the electron difference map and refined with their thermal parameters linked to their parent atoms. The positions of the remaining C-H atoms were calculated geometrically and refined using the riding model.

_refine_special_details: The crystal is a merohedral twin; an appropriate twin law has been applied to the refinement. The disordered DMSO solvent molecules have been modelled over two or three positions, using thermal and geometric parameter restraints (Figure S14).

_exptl_absorpt_process_details: **CrysAlisPro** 1.171.40.47a (Rigaku Oxford Diffraction, 2019). Numerical absorption correction based on Gaussian integration over a multifaceted crystal model. Empirical absorption correction used spherical harmonics as implemented in SCALE3 ABSPACK.

The value of Z is 4 and Z' is 2. There are two independent molecules and four solvent DMSOs in the asymmetric unit (Figure S15).

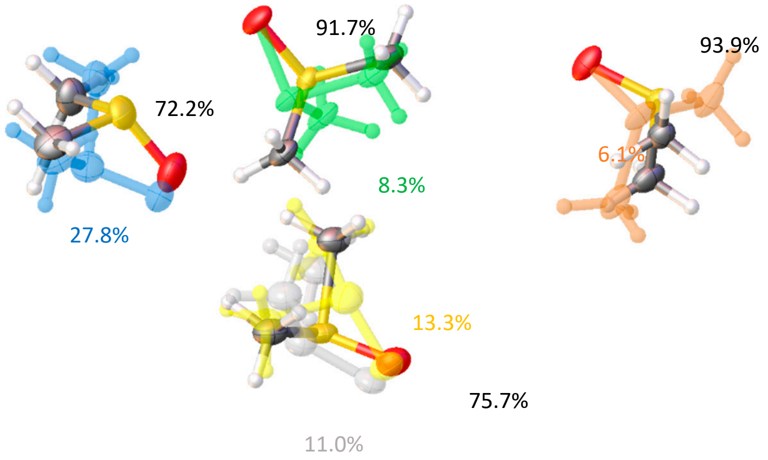

Figure S14. The disordered DMSO solvent molecules within **A**, thermal ellipsoids drawn at the 50% probability level, minor component(s) were drawn with ghosted colours.

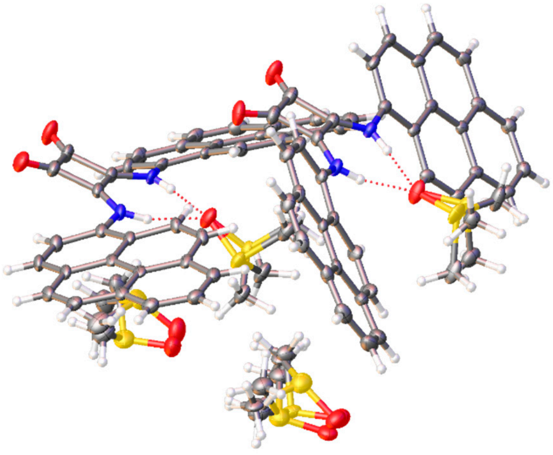

Figure S15: The asymmetric unit of **A**, thermal ellipsoids drawn at the 50% probability level, disorder shown.

2) Structure (B): $H_2L \cdot Cl^- \cdot TBA^+ \cdot 3(TBA^+Cl^-) \cdot 7.5\ H_2O$

Crystal Data. $C_{100}H_{179}Cl_4N_6O_{9.5}$, M_r = 1759.28, triclinic, *P*-1 (No. 2), a = 15.9447(3) Å, b = 17.8943(3) Å, c = 18.6302(3) Å, = 88.6300(10)°, = 80.1610(10)°, = 89.475(2)°, *V* = 5235.81(16) Å^3, *T* = 100(2) K, *Z* = 2, *Z'* = 1, (MoK) = 0.168 mm^{-1}, 121234 reflections measured, 23977 unique (R_{int} = 0.0352) which were used in all calculations. The final wR_2 was 0.1629 (all data) and R_1 was 0.0582 (I > 2(I)).

X-ray data collected upon a yellow (cut) lath-shaped crystal (0.198×0.047×0.030 mm^3), mounted on a MITIGEN holder with perfluoroether oil; using a Rigaku FRE+ diffractometer, equipped with Varimax confocal mirrors, an AFC12 goniometer, a HyPix 6000 detector and an Oxford Cryosystems low-temperature device, operating at *T* = 100(2) K.

Data measured using profile data from -scans of 0.5 ° per frame for 19.0 s using Mo K radiation (Rotating Anode, 45.0 kV, 55.0 mA). The total number of runs and images based on the strategy calculation from the program **CrysAlisPro** (Rigaku, V1.171.41.89a, 2020). The maximum resolution achieved was = 27.486°.

Cell parameters were retrieved using **CrysAlisPro** (Rigaku, V1.171.41.89a, 2020) and refined using 39987 reflections, 33% of the observed reflections. Data reduction was performed using **CrysAlisPro** (Rigaku, V1.171.41.89a, 2020), which corrects for Lorentz polarisation. The final completeness is 99.90 % (**IUCr**) out to 27.486° in .

A Gaussian absorption correction was performed using **CrysAlisPro** (Rigaku, V1.171.41.89a, 2020). Numerical absorption correction was based on Gaussian integration over a multifaceted crystal model. Empirical absorption correction using spherical harmonics, implemented in SCALE3 ABSPACK scaling algorithm. The absorption coefficient of this material is 0.168 mm^{-1} at this wavelength (= 0.71075 Å) and the min/max transmissions are 0.736 and 1.000.

The structure was solved in the space group *P*-1 (# 2) by using dual methods using **ShelXT** 2018/2 (Sheldrick, 2015) and refined by full matrix least squares minimisation on F^2 using **ShelXL** 2018/3 (Sheldrick, 2015). All non-hydrogen atoms were refined anisotropically. The positions of N-H atoms H1 and H2 were located from the electron difference map and refined with their thermal parameters

linked to their parent atoms; with the positions of the solvent water O-H and all the remaining C-H atoms, calculated geometrically and refined using the riding model.

_refine_special_details: This sample contained both block-like and plate-like yellow crystals; this data is from a representative block-like crystal. The disordered atoms of the TBA ions (C39a/C39b, C40a/C40b, C43a/C43b, C44a/C44b, C54a/C54b > C56a/C56b, and C58a/C58b > C60a/C60b) are modelled over two positions using thermal and geometric parameter restraints and the disordered waters/chloride ion sites, are modelled using thermal parameter restraints. The occupancy ratio between these water/chloride ion sites is freely refined very close to 1:1; therefore, for all subsequent refinements this ratio was fixed at 0.5:0.5. In addition, all of the water molecules were modelled and refined as rigid bodies with idealised geometries. Applying the above to the refinement, conserved realistic chemical geometries and lowered R_1 from 7.67% to 5.82%.

_exptl_absorpt_process_details: **CrysAlisPro** (Rigaku, V1.171.41.89a, 2020). Numerical absorption correction was based on Gaussian integration over a multifaceted crystal model. Empirical absorption correction used spherical harmonics, implemented in SCALE3 ABSPACK scaling algorithm.

Z is 2 and Z' is 1. There is a single ligand molecule, four chloride ions, four TBA ions and seven solvent water molecules in the asymmetric unit, represented by the reported sum formula.

References

CrysAlisPro Software System, Rigaku Oxford Diffraction, (2020).

O.V. Dolomanov and L.J. Bourhis and R.J. Gildea and J.A.K. Howard and H. Puschmann, Olex2: A complete structure solution, refinement and analysis program, *J. Appl. Cryst.*, (2009), **42**, 339-341.

Sheldrick, G.M., Crystal structure refinement with ShelXL, *Acta Cryst.*, (2015), **C71**, 3-8.

Sheldrick, G.M., ShelXT-Integrated space-group and crystal-structure determination, *Acta Cryst.*, (2015), **A71**, 3-8.
